# Inhaled antibiotics for treating pneumonia in invasively ventilated patients in intensive care unit: a meta-analysis of randomized clinical trials with trial sequential analysis

**DOI:** 10.1186/s13054-024-05159-9

**Published:** 2024-11-25

**Authors:** Nicolò Sella, Tommaso Pettenuzzo, Alessandro De Cassai, Francesco Zarantonello, Sabrina Congedi, Andrea Bruni, Eugenio Garofalo, Honoria Ocagli, Dario Gregori, Federico Longhini, Paolo Navalesi, Annalisa Boscolo, the PADOVA ICU Group, Carlo Albero Bertoncello, Nicola Franchetti, Chiara Schiavolin, Giuliana Carofiglio, Chiara Guidotto, Giovanni De Lorenzi, Christian Legnaro, Marco Nardelli, Elisa Pistollato, Giulia Mormando, Arianna Peralta, Enrico Petranzan, Luisa Muraro, Paolo Persona, Giorgia Pacchiarini

**Affiliations:** 1 Institute of Anesthesia and Intensive Care, Padua University Hospital, Padua, Italy; 2 Department of Medicine (DIMED), Section of Anaesthesiology and Intensive Care, University of Padua, 13, Vincenzo Gallucci Street, 35125 Padua, PD Italy; 3 Department of Medical and Surgical Sciences, Magna Graecia University, Catanzaro, Italy; 4 Unit of Biostatistics, Epidemiology and Public Health, Department of Cardiac, Thoracic, Vascular Sciences and Public Health, University of Padova, Padua, Italy; 5 Department of Cardiac, Thoracic, Vascular Sciences and Public Health, University of Padua, Padua, Italy

**Keywords:** Antibiotics, Inhaled, Nebulized, Treatment, Infection, Multi-drug resistant, Multi-drug resistant organism

## Abstract

**Background:**

The use of inhaled antibiotics for treating pneumonia in invasively ventilated patients offers a direct approach, allowing for high local concentrations of the drug in the lower respiratory tract while simultaneously reducing systemic toxicity. However, the real efficacy and safety of nebulized antibiotics remain unclear. The aim of the present is to assess among critically adult patients with pneumonia and invasive ventilation, whether receiving adjuvant inhaled antibiotics improves the rate of microbiological eradication.

**Methods:**

A comprehensive literature search of randomized clinical trials (RCTs) was conducted (from inception until September 20, 2024, PROSPERO-CRD592906) across Medline, Embase, and Scopus. Randomized controlled trials, enrolling intensive care units (ICU) patients with pneumonia and comparing nebulized antimicrobial therapy (inhaled group) with intravenous antimicrobial treatment or intravenous antimicrobial therapy plus inhaled placebo (control group), were included. The primary outcome was the rate of microbiological eradication after treatment. Secondary outcomes were the rate of clinical recovery, the incidence of drug-related adverse events, ICU and hospital mortality. A qualitative analysis was conducted according to the GRADE framework. Data were pooled using an odds-ratio analysis. The heterogeneity and reliability of our results were evaluated using the I^2^-statistic and trial sequential analysis (TSA), respectively.

**Results:**

A total of 11 RCTs (1472 patients) met the inclusion criteria. Compared to controls, the use of adjuvant inhaled antibiotics determined a greater rate of microbiological eradication (OR 2.63, 95% CI 1.36–5.09; low certainty of evidence). The TSA confirmed the reliability of our primary outcome. Moreover, nebulized antibiotics increased the risk of bronchospasm (OR 3.15, 95% CI 1.33–7.47; high evidence), while nephrotoxicity, clinical recovery, ICU and hospital survival (either in the case of pneumonia caused by MDR bacteria or not) were not different between groups.

**Conclusions:**

In conclusion, compared to the sole intravenous therapy, the use of adjuvant inhaled antibiotics for treatment of pneumonia in invasively ventilated critically ill patients was associated with a greater incidence of microbiological eradication (low GRADE and high risk of publication bias), but not with clinical recovery and survival.

**Supplementary Information:**

The online version contains supplementary material available at 10.1186/s13054-024-05159-9.

## Background

Pneumonia is one of the most frequent infections among critically ill patients in intensive care units (ICUs) [1], accounting for 65% of the infections registered at the time of ICU admission in a large multicenter study [2]. Furthermore, up to 40% of patients under invasive mechanical ventilation (IMV) for more than 48 h develop ventilator-associated pneumonia (VAP) [3], with an average rate of VAP ranging between 1 and 2.5 cases/1000 days of IMV in the US [4] and 8.9 cases/1000 days of IMV in Europe [3]. Such a high prevalence is associated with an alarming burden of mortality, being pneumonia associated with a mortality rate of 34–44% in an American epidemiological study in more than 8 million of mechanically ventilated patients [5]. Moreover, pneumonia has also been associated with increased IMV duration, longer ICU and hospital stays, and higher healthcare costs [3].

Several international guidelines have been established for the management of pneumonia [6–9], in general suggesting early initiation of empirical broad spectrum antibiotic treatment, followed by focused and narrowed antimicrobial therapy according to the results of microbiological analysis, especially samples of the lower respiratory tract. However, even when optimal care is applied according to the guidelines recommendations, the risk of unsuccessful treatment remains consistent, especially among critically ill patients, in whom the rate of treatment failure has been reported to be as high as 31–64% [10–12]. These disappointing results have been related both to the increased prevalence of difficult-to-treat and multidrug-resistant (MDR) bacteria in the ICU population, and to the severe pathophysiological alterations of the critically ill patients that can affect antibiotic pharmacokinetics [3, 8, 9, 13–15]. In fact, in critical illness the dysfunction of several organ systems leads to a significant pharmacokinetic variability and the plasma concentration of antibiotics may be either reduce or increased, mainly due to the complex interaction of multiple factors, such as fluid overload, hypoalbuminemia, altered protein binding, hyperdynamic state, tissue hypoperfusion, renal and liver failure, and extracorporeal organ support [16]. In the case of severe pneumonia, reaching an effective antibiotic concentration in the injured lung is even more challenging, since after systemic administration antimicrobial drugs must cross the alveolar capillary barrier. The cross of the alveolar capillary barrier depends both on the physicochemical and pharmacokinetic characteristics of the antibiotic, as well as on the anatomopathological characteristics of the patient’s disease, which can alter the normal alveolar capillary barrier [16, 17].

For these reasons, inhaled administration of antimicrobial drugs has been proposed to increase antibiotic concentrations within the affected lung while minimizing systemic exposure [18]. Despite promising premises, the use of nebulized antibiotics for pneumonia treatment in critically ill patients remains controversial. The 2016 Clinical Practice Guidelines of the Infectious Diseases Society of America and the American Thoracic Society support the use of nebulized antibiotics as an adjunctive treatment with intravenous drug for patients with VAP due to Gram-negative bacteria that are susceptible to only aminoglycosides or polymyxins, or as a last resort treatment for patients who do not respond to intravenous antibiotics alone, regardless of whether the pathogen is MDR [8]. However, one multicenter, randomized, double-blind, placebo-controlled trial failed to show any survival benefits of adjunctive aerosolized antibiotics for ICU patients with suspected MDR Gram-negative pneumonia [19]. Since the complexity of critically ill patients makes it difficult to attribute death to the treatment under investigation with certain degree of causality, mortality has been questioned as primary outcomes in several RCTs, while intermediate events have been valued in order to tighten the coupling of intervention and outcome, to reduce the potential contamination from other factors, and finally to better reflect the therapeutic intent of treatment in the complex clinical realities of the ICU [20]. Therefore, we designed the present systematic review and meta-analysis of randomized controlled trials (RCT), aiming to assess among critically ill adult patients, invasively ventilated and affected by pneumonia (P), whether receiving nebulized antibiotics as adjunctive treatment (inhaled group) (I), compared to being treated only with intravenous antimicrobial therapy or with intravenous antimicrobial therapy plus inhaled placebo (control group) (C), results in different short- and long-term clinical outcomes (i.e., microbiological eradication (our primary outcome, defined as the complete elimination of a specific microorganism from patients’ airways), clinical recovery, drug-related adverse events, ICU and hospital survival) (O).

## Methods

The Preferred Reporting Items for Systematic reviews and Meta-Analysis (PRISMA) Statement was followed for writing this systematic review and meta-analysis (Additional Material 1) [21]. The review protocol was registered in PROSPERO (CRD592906), an international prospective registry of systematic reviews.

### Literature search

An electronic search of Medline, Embase, and Scopus from inception until September 20, 2024 was performed with no language restrictions. Furthermore, grey literature (OpenGrey) and all references of included articles and related reviews and guidelines were searched. A full description of search strategies is reported in Additional Material 2.

### Study selection and data collection

All studies meeting the following Participants, Interventions, Comparisons, Outcomes, and Study design (PICOS) questions were included: participants were adult patients with pneumonia admitted to the ICU and receiving IMV; the intervention was inhaled antimicrobial therapy as an adjunctive treatment (inhaled group); the comparison was intravenous antimicrobial therapy or intravenous antimicrobial therapy plus inhaled placebo (control group); the primary outcome was the rate of microbiological eradication, while clinical recovery, drug-related adverse events, ICU and hospital survival were secondary outcomes [22–24]; eligible study designs were RCTs. Search results were merged and duplicate records from the same report were removed.

**Fig. 1:**
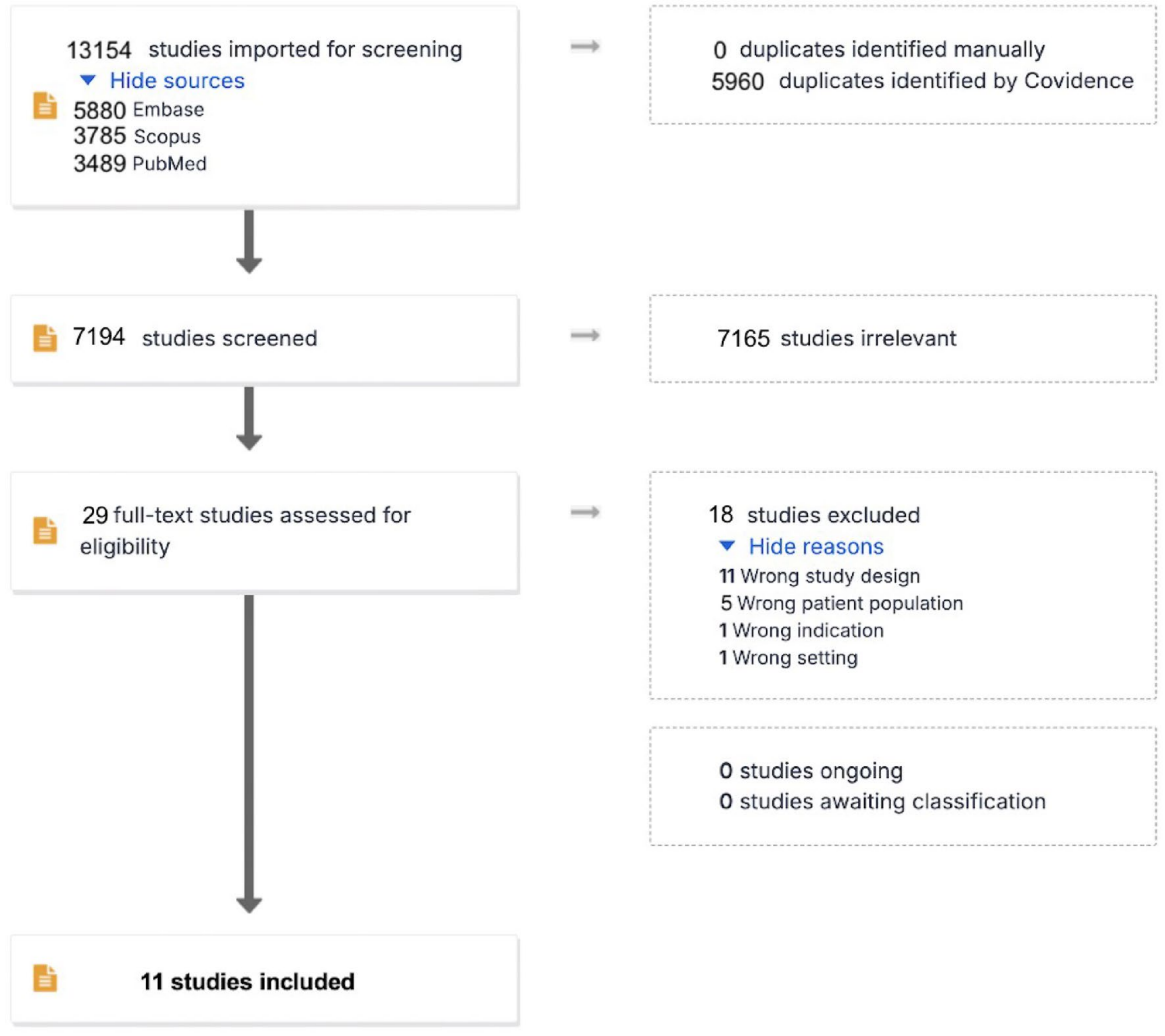
PRISMA flow-chart

Six researchers (PT, SN, DCA, CS, BrA and BA) were split into three couples, each analyzing the same number of overall identified citations. Specifically, each member of the couple independently screened the titles and abstracts of the assigned papers and retrieved the full texts of potentially relevant reports. The reasons for exclusion are reported in Fig. 1. Covidence systematic review software (Veritas Health Innovation, Melbourne, Australia; available at www.covidence.org) was used for study selection.

Four researchers (PT, SN, DCA, and CS) were split into two couples, each analyzing the same number of eligible full texts. Specifically, each member of the couple independently assessed the full text of the assigned papers.

Data from included studies were recorded using a Microsoft Excel (Microsoft Corporation, Redmond, WA, USA) report form. Two researchers (BrA and BA) independently verified all extracted data for accuracy. Any disagreements on study selection and data extraction were resolved by referral to other authors (NP or GE), if necessary.

The following information was collected: first author, study year, journal, type of patients included (i.e., medical and/or surgical), number of patients and their baseline characteristics, device for providing nebulized antimicrobial therapy, inclusion and exclusion criteria, type of antimicrobials, outcomes of interest (Additional Material 3).

### Certainty of evidence assessment

Four researchers (GE, NP, BA and DCA) were split into two couples and assessed the risk of bias of the same number of included studies. Specifically, each member of the couple independently evaluated the quality of included RCTs using the Risk of Bias (RoB) 2 assessment tool, which examines five domains of bias, i.e., the randomization process, deviations from intended interventions, missing outcome data, outcome measurement, and selection of the reported results. The RoB 2 tool categorizes the study-level risk of bias on a three-grade scale, i.e., low risk of bias, high risk of bias, or some concerns [25, 26]. Disagreements were resolved by discussion with another author (TP), if necessary.

Publication bias was evaluated by visually inspecting a funnel plot for potential asymmetry and Egger’s test was applied when the number of studies was greater than 10 (www.training.cochrane.org/handbook.).

The Grades of Recommendation, Assessment, Development and Evaluation (GRADE) approach was applied to assess the certainty of evidence related to the primary outcomes and some secondary outcomes [27].

### Subset analyses

Additional analyses were conducted to assess the impact of inhaled antibiotics on the primary outcome considering: (i) studies exclusively enrolling medical populations or mixed cohorts (i.e., including medical and surgical patients); (ii) studies employing different classes of inhaled antibiotics (i.e., aminoglycoside or polymyxin); and (iii) studies using different devices for the administration of nebulized antimicrobial therapy (i.e., vibrating mesh or nebulizer).

### Sensitivity analysis

We evaluated the robustness of our primary outcome distinguishing between studies with different risks of bias assessment and, by removing one paper at a time from the analysis. Furthermore, we performed a post-hoc sensitivity analysis considering one of the two inhaled antibiotic groups described by Ammar et al. [28].

### Post-hoc trial sequential analysis

We performed a Trial Sequential Analysis (TSA) with a type I error rate of 5%, 90% power, and a clinically significant difference on microbiological eradication between intervention and control of 50%. The two-sided α-spending boundaries and the futility area were calculated with the O’Brien-Fleming function [29].

### Post-hoc analysis

Moreover, we performed a post-hoc subgroup analysis on the rate of microbiological eradication, clinical recovery, ICU and hospital survival comparing studies exclusively enrolling patients with pneumonia due to pre-detected MDR bacteria to those RCTs including also pneumonia due to multisensitive bacteria. Finally, despite a high fragility index, we analyzed the impact of inhaled antimicrobial therapy on the overall antibiotic duration.

### Statistical analysis

The treatment effect for continuous outcomes was analyzed with the inverse variance method and expressed as mean difference (MD) or standardized mean difference (SMD) with 95% confidence interval (CI), as appropriate. The treatment effect for dichotomous outcomes was analyzed using the Mantel–Haenszel method and expressed as odds ratio (OR) with 95% CI. Where necessary, we converted the reported median and interquartile range or the first-third quartile to estimated mean and standard deviation (SD) using Hozo's method [30]. We applied a continuity correction in the case where there are no events in the groups.

To assess statistical heterogeneity, we used the Chi-squared test and the I^2^ -statistic, categorizing heterogeneity values as follows: low (I^2^ < 25%), moderate (I^2^ between 25 and 50%), and high (I^2^ > 50%) [31]. We consistently opted for a random-effects model, regardless of the heterogeneity level, to account for heterogeneity between studies.

All analyses were performed with R version 4.3.3 (The R Foundation for Statistical Computing, Vienna, Austria) with the “meta” and “metafor” packages and the Trial Sequential Analysis software (version 0.9.5.10, Copenhagen Trial Unit, Centre for Clinical Intervention Research, Copenhagen). For all analyses, two-sided *p* values < 0.05 were considered significant.

## Results

### Study selection and data retrieval

The PRISMA flowchart, shown in Fig. 1, illustrates the study selection process. Initially, 13,154 studies were retrieved from databases. Additional consultations with experts and authors of the included articles did not produce any further study for evaluation beyond those papers already identified. At the end of the process, 11 RCTs entered qualitative and quantitative analysis [11, 19, 22, 24, 28, 32–37]. We requested missing data (twice) from all authors of the included trials, but none was able to provide the missing information.

### Study characteristics

The 11 included studies comprised a total of 1472 patients (767 (52%) assigned to the inhaled group and 705 (48%) to the control group). The characteristics of all studies are briefly reported in Additional Material 3.

**Fig. 2:**
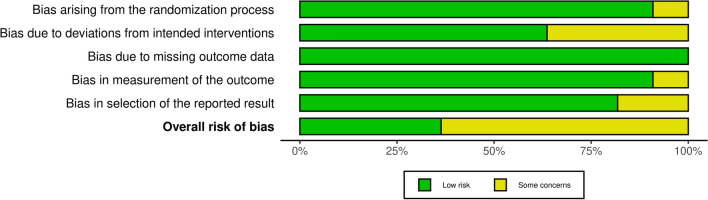
Manuscript quality assessment using risk of bias (ROB)-2 tool

According to the risk of bias evaluation: four studies were at low risk of bias [11, 19, 32, 37], while seven studies raised some concerns [22, 24, 28, 33–36] (Fig. 2). The rationale for each RoB judgment is provided in Additional Material 4.

### Primary outcome

**Fig. 3:**
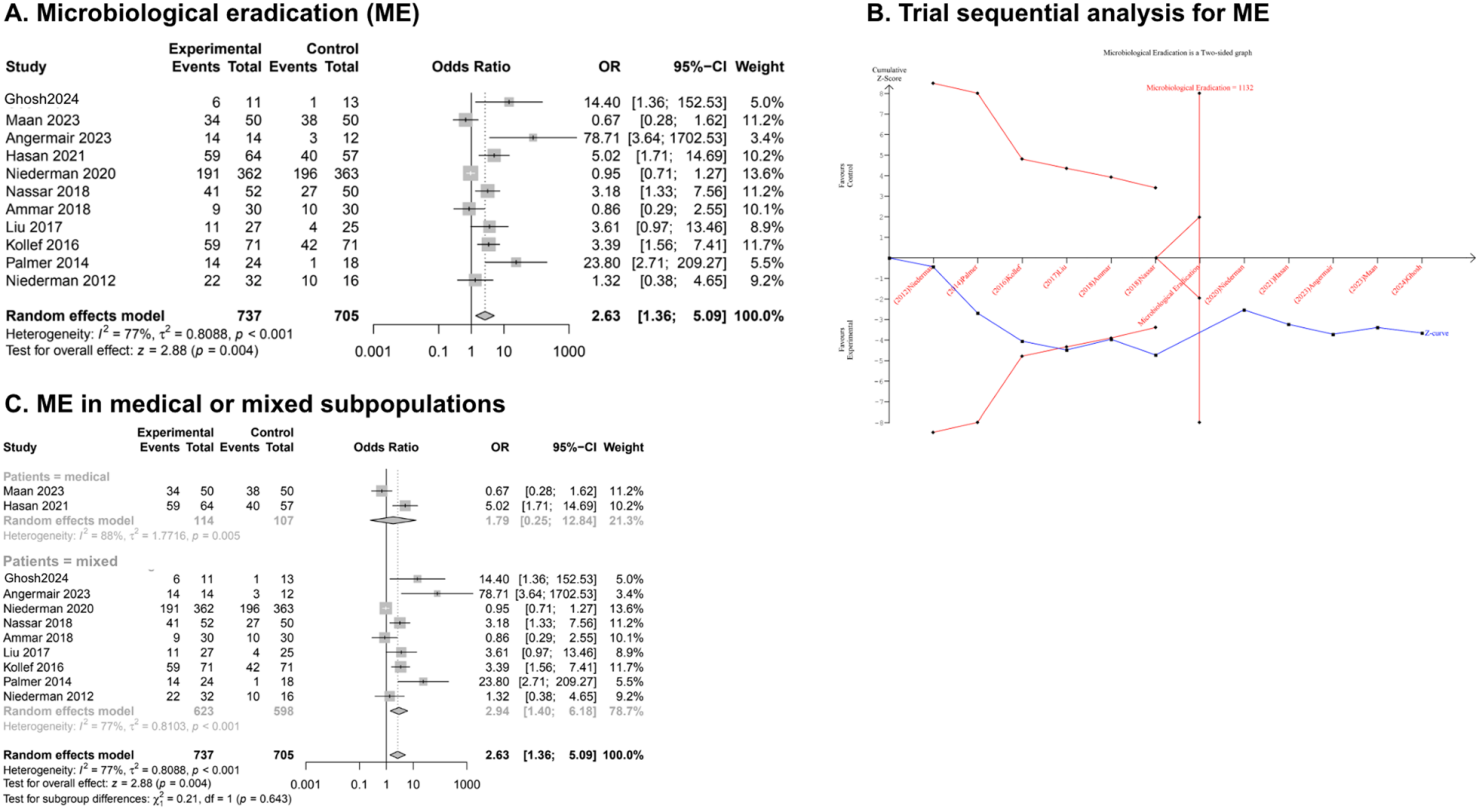
Primary outcome and TSA. *Abbreviations:* OR, odds ratio; CI, confidential interval; TSA, trial sequential analysis

The overall rate of microbiological eradication, reported by 11 RCTs [11, 19, 22, 24, 28, 32–37], was greater in the inhaled group (OR 2.63, 95% CI 1.36–5.09, I^2^ 77%; low certainty of evidence), as compared to controls (Fig. 3A-B, Table 1). According to the subset analysis, the main significance was obtained from studies enrolling mixed (i.e., surgical and medical) patients (OR 2.94, 95% CI 1.40–6.18, I^2^ 77%), and not exclusively medical subjects (OR 1.79, 95% CI 0.25–12.84, I^2^ 88%). However, the test for subgroup differences was not significant (*p* value 0.643) (Fig. 3C).

**Table 1:** Grades of recommendation, assessment, development and evaluation summary of findings table

Certainty assessment							Summary of findings		
Participants (studies)	Risk of bias	Inconsistency	Indirectness	Imprecision	Other considerations	Overall certainty of evidence	Inhaled antibiotics group (N/total)	Control group (N/total)	Relative effect as OR or MD, (95% CI) of inhaled antibiotics
Rate of microbiological eradication									
1442 (11 RCTs)	Serious^a^	Serious^b^	Not serious	Not serious	Publication bias strongly suspected^c^ Strong association^d^	⨁⨁◯◯ LOW	464/737 (63.0%)	368/705 (52.2%)	2.63 (1.36–5.09)
ICU survival									
1394 (10 RCTs)	Serious^a^	Not serious	Not serious	Not serious	None	⨁⨁⨁◯ MODERATE	475/705 (67,4%)	441/689 (64,0%)	1.34 (0.93–1.95)
Hospital survival									
1035 (5 RCTs)	Not serious	Serious^e^	Not serious	Serious^f^	None	⨁⨁◯◯ LOW	299/520 (57,5%)	289/515 (56,1%)	1.19 (0.56–2.52)
Clinical recovery									
373 (5 RCTs)	Serious^g^	Serious^h^	Not serious	Serious^f^	None	⨁◯◯◯ VERY LOW	92/190 (48.4%)	85/183 (46.4%)	1.40 (0.49–4.00)
Bronchospasm									
948 (5 RCTs)	Serious^i^	Not serious	Not serious	Not serious	Strong association^d^	⨁⨁⨁⨁ HIGH	24/478 (5.0%)	7/470 (1.5%)	3.15 (1.33–7.47)
Nephrotoxicity									
1050 (7 RCTs)	Serious^j^	Not serious	Not serious	Serious^f^	None	⨁⨁◯◯ LOW	51/532 (9.6%)	60/518 (11.6%)	0.83 (0.56–1.24)

N: number of patients; OR: odds ratio; MD: mean difference; CI: confidence interval; ICU: intensive care unit; RCT: randomized controlled trials; MDR: multi-drug resistant

“Other considerations” include publication bias, large effect, plausible confounding, and dose response gradient

^a^Seven studies arose some concerns, ^b^Large unexplained inconsistency (I^2^ = 77%, τ^2^ = 0.8088, *p* < 0.001), ^c^Publication bias was strongly suspected from the visual inspection of the funnel plot and the accompanying Egger’s regression (*p* = 0.001), ^d^Large intervention effect (OR > 2.0), ^e^Large unexplained inconsistency (I^2^ = 68%, τ^2^ = 0.4565, *p* = 0.015), ^f^Confidence interval including appreciable benefit or harm, ^g^Four studies arose some concerns, ^h^Large unexplained inconsistency (I^2^ = 75%, τ^2^ = 1.0367, *p* = 0.003), ^i^Four studies arose some concerns, ^j^Five studies arose some concerns

Similarly, no subgroup differences were found comparing inhaled aminoglycosides (OR 2.68, 95% CI 1.08–6.63, I^2^ 77%) to polymyxins (OR 2.83, 95% CI 0.89–9.01, I^2^ 76%) (*p* value for subgroup differences = 0.942) (Additional Material 5A); or different type of device used for nebulization (*p* value for subgroup differences = 0.781) (Additional Material 5B). Moreover, the sensitivity analysis further corroborated our findings (Additional Materials 6 and 7). To note, studies with some concerns of risk of bias showed a greater rate of microbiological eradication in the inhaled group (OR 2.98, 95% CI 1.18–7.52, I^2^ 71%); while studies at low risk of bias showed no differences between subgroups (OR 2.38, 95% CI 0.78–7.25, I^2^ 82%), despite a *p* value for subgroup differences not significant (*p* value 0.761) (Additional Materials 6). Finally, the TSA confirmed the adequacy of the current sample size and the reliability of our primary outcome (Fig. 3B).

### Secondary outcomes

**Fig. 4:**
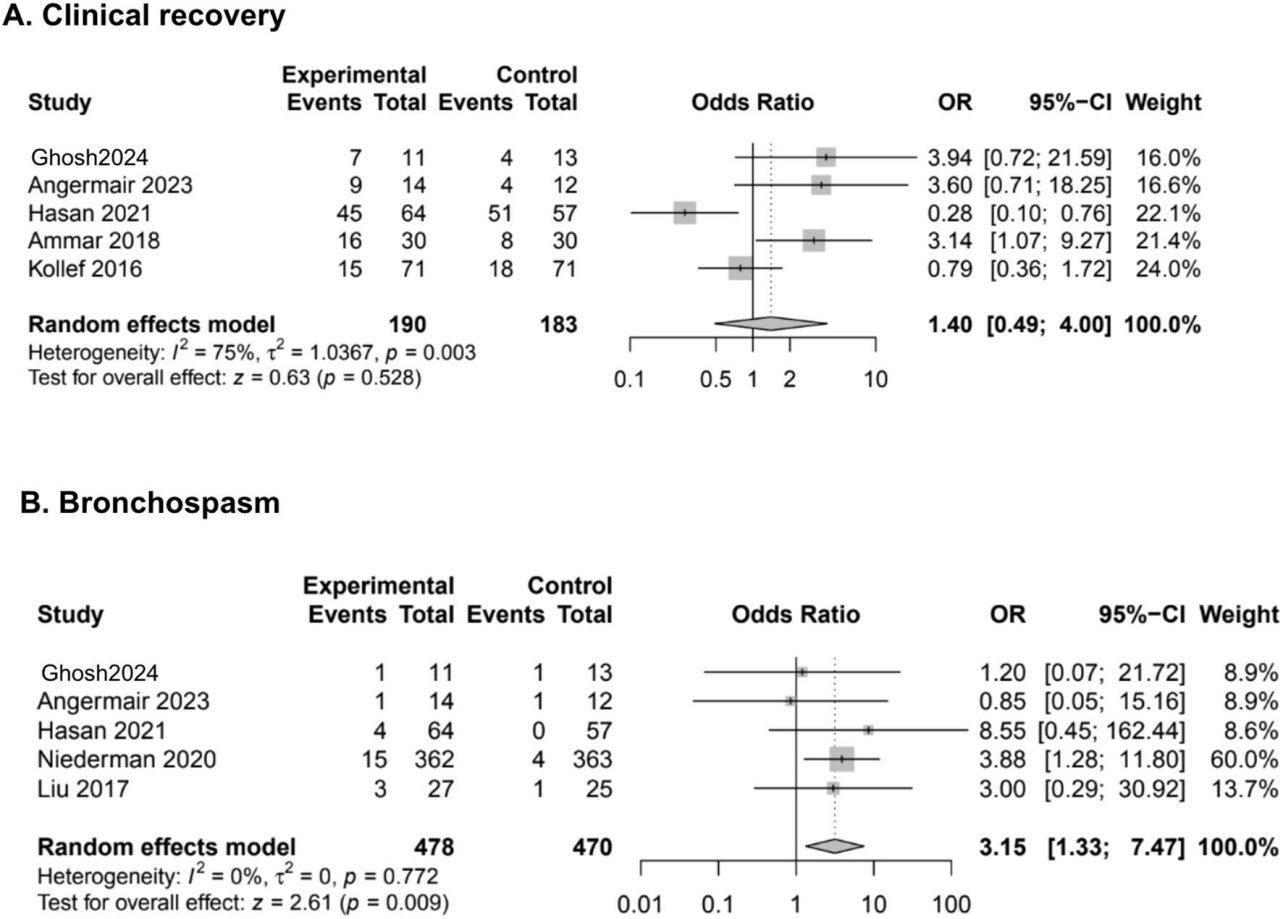
Most relevant secondary outcomes. *Abbreviations:* OR, odds ratio; CI, confidential interval

Overall, the rate of clinical recovery [11, 24, 28, 35, 36] was similar between the inhaled and control group (OR 1.40, 95% CI 0.49–4.00, I^2^ 75%) (very low certainty of evidence) (Fig. 4A, Table 1).

The risk of bronchospasm, described by 5 studies [19, 24, 34–36], was greater in the inhaled group, as compared to controls (OR 3.15, 95% CI 1.33–7.47, I^2^ 0%) (high certainty of evidence) (Fig. 4B, Table 1). However, the overall incidence of this specific adverse event was low (less than 5%) and, consequently, the fragility index of this finding was relatively high.

Conversely, nephrotoxicity, investigated by 7 studies [19, 24, 28, 32, 34–36], was similar between cases and controls (OR 0.83, 95% CI 0.56–1.24, I^2^ 0%) (low certainty of evidence) (Additional Material 8A, Table 1). Similarly, ICU survival, reported by 10 RCTs [11, 19, 22, 24, 28, 32–36] (OR 1.34, 95% CI 0.93–1.95, I^2^ 7%; moderate certainty of evidence), and hospital survival, described in 5 RCTs [11, 19, 32, 33, 36] (OR 1.19, 95% CI 0.56–2.52, I^2^ 68%; low certainty of evidence), were comparable between the two groups (Additional Material 8B and C, Table 1).

### Post-hoc analysis

Comparing studies enrolling pneumonia exclusively due to MDR bacteria to those RCTs including also patients with multisensitive bacteria, microbiological eradication, clinical recovery, ICU and hospital mortality were similar between different subpopulations (Additional Material 9 A-D). Finally, additional data were reported in Additional Material 10.

### Publication *bias*

Regarding publication bias, only microbiological eradication and ICU survival had sufficient studies to allow for the execution of the Egger test, which revealed a significant publication bias only considering the primary outcome (*p* = 0.001 and *p* = 0.436, respectively). For the other outcomes, visual inspections were conducted across all results, with mild asymmetries identified only considering clinical recovery (Additional Material 11A-F).

## Discussion

The present systematic review and meta-analysis of 11 RCTs, in invasively ventilated patients with (mainly nosocomial and ventilator-associated) pneumonia, shows that, compared to only intravenous antimicrobial therapy, adjuvant inhaled antibiotics determined a greater rate of microbiological eradication, and the TSA confirmed the reliability of our primary outcome. Noteworthy, inhaled antibiotics increased the risk of bronchospasm, while not of nephrotoxicity. Finally, clinical recovery and, in particular, ICU and hospital survival, as anticipated by previous literature [38–41], were similar between inhaled and control groups (also in case of pneumonia exclusively due to MDR bacteria).

In keeping with previous meta-analysis, on the efficacy and safety of antibiotic nebulization in mechanically ventilated patients and including RCTs and prospective or retrospective observational studies [42, 43], our updated study suggested that the administration of nebulized antibiotics could increase the probability of microbiological eradication, despite a missing effect on the incidence of clinical recovery, not completely in line with the results reported by Xu et al. [43], while more similar to Candela Solé-Lleonart’s findings [42]. These discrepancies could be related to the fact that microbiological eradication could not necessarily equate to clinical success. In fact, many other clinical and patient-related factors, not considered in our analysis, may affect clinical success. Second, the previous meta-analysis [42, 43] were characterized by a high intra- and inter-studies heterogeneity, where the inclusion also of observational studies and trials on milder diseases, such as ventilator-associated tracheobronchitis, led to more optimistic results, decreasing the incidence of VAP and improving clinical recovery [43]. Infact, our meta-analysis included only RCTs, for limiting potential inter-studies dishomogeneity and avoiding the risk of selection bias. For instance, Palmer et al. [44] was excluded because the authors enrolled also tracheobronchitis, potentially more responsive to aerosolized therapies; while Lu et al. was removed because the authors used inhaled antibiotics as an alternative antimicrobial treatment, and not as an adjunctive therapy to the systemic antimicrobial drugs [45].

Consistent with the result of the largest and most recent RCT on this topic [19], investigating the impact of nebulized amikacin adjunctive to intravenous standard-of-care antibiotics on 28–32 day survival, our study confirms that aerosolized antibiotics had no benefits both on ICU and hospital mortality, regardless the incidence of MDR bacteria. However, these specific outcomes, especially hospital mortality, can be influenced by numerous clinical and patient-related factors that may not have been considered in our analysis [20, 38–41].

Additionally, despite a similar risk of nephrotoxicity [46–48], our meta-analysis suggests that inhaled antibiotics increase the rate of local complications, particularly bronchospasm. These findings advocate a careful clinical evaluation before administering inhaled antibiotics in critically ill patients, thoughtfully balancing benefits and risks of potential adverse events [48].

Focusing on the clinical scenarios that could benefit more from nebulized antibiotics, further studies are need to evaluate if these treatments may be reserved to specific subpopulations, such as solid organ transplant recipients or patients requiring extracorporeal life support or prolonged IMV, who are particularly at risk of developing MDR strains and for whom the occurrence of difficult-to-treat infections could significantly affect survival [14, 15]. In fact, in these specific clinical scenarios, the use of nebulized antibiotics could be more promising in prevention rather than in treatment [49]. However, we found that aerosolized adjuvant antibiotics had similar effect in mixed and medical populations in order to improve microbiological eradication.

Moreover, focusing on the choice of the antimicrobial drug, our data suggest that aminoglycoside and polymyxin are similarly useful in reaching microbiological eradication and, in addition, no differences were recorded based on the type of device used for aerosolization.

Our meta-analysis has some strong points. Compared to all previously published reviews on the topic [42, 43, 50], our findings are derived exclusively from RCTs involving mechanically ventilated patients, while other study designs were not considered. Furthermore, TSA showed that the primary outcome of microbiological eradication after therapy achieved the required sample size, thereby reinforcing the robustness of our findings. Finally, we also included the most recent trials, especially those published after 2014. In fact, in 2014 the recommended doses of colistin were increased markedly [51, 52] according to pharmacokinetics/pharmacodynamics studies [53], therefore, trials conducted before 2014 may have used infra-therapeutic doses of antibiotics.

We would not be remiss to mention some limitations of our work. First, the number of included studies is limited. Only 11 RCTs described our primary outcome. This limitation is due to the strict criteria for study selection. Indeed, only RCTs, involving invasively mechanically ventilated patients with pneumonia, were included, while trials with colonized but not infected patients and non-RCT studies were excluded. This harsh selection, despite reducing the number of eligible studies, makes our results solid and generalizable to the specific study population. Second, microbiological eradication, particularly when achieved through inhaled antibiotics in ventilated patients, is indeed an anticipated outcome, since systemic antibiotics often fail to eradicate bacteria from protected areas such as biofilms in the airway. However, microbiological eradication was the outcome with the largest number of available RCTs and, we preferred to focus our attention on this primary outcome, rather than on mortality, because patients’ survival depends on a paramount of additional clinical factors and confounders [38–41], not adequately analyzed in our study. Indeed, microbiological eradication is only the initial step of a much more in-depth analysis of some possible repercussions of inhaled antibiotics on the clinical course of patients with pneumonia.

Third, different definitions for microbiological eradication, clinical recovery, bronchospasm, and nephrotoxicity in the included trials could affect the robustness of our findings. Four, an important limitation of this analysis and these RCTs is the lack of assessment on duration of treatment. In theory, inhaled antibiotics should be able to reduce the overall duration of antibiotic administration for pneumonia. Further studies are necessary to investigate the real impact of inhaled antibiotics on the overall duration of antibiotics for treating pneumonia.

Finally, the certainty of evidence was relatively low in some outcomes of interest and the risk of publication bias for the primary outcome was significant, limiting the strength of our findings and confirming the need for further larger-scale RCTs.

## Conclusions

In conclusion, compared to the sole intravenous therapy, the use of adjuvant inhaled antibiotics for treatment of pneumonia in invasively ventilated critically ill patients was associated with a greater incidence of microbiological eradication (low GRADE and high risk of publication bias) but not with clinical recovery and survival.

## Supplementary Information

Additional file 1.

## Data Availability

No datasets were generated or analysed during the current study.

## Abbreviations

CI: Confidence interval
GRADE: Grades of Recommendation, Assessment, Development and Evaluation
ICU: Intensive case unit
IMV: Invasive mechanical ventilation
OR: Odds ratio
MD: Mean difference
MDR: Multi-drug resistant
MDRO: Multi-drug resistant organism
PRISMA: Preferred Reporting Items for Systematic reviews and Meta-Analysis
RCT: Randomized controlled trial
RoB: Risk of bias
SD: Standard deviation
TSA: Trial sequential analysis
VAP: Ventilator-associated pneumonia
