# Supplementary material for: Inhaled antibiotics for treating pneumonia in invasively ventilated patients in intensive care unit: a meta-analysis of randomized clinical trials with trial sequential analysis
Source: Crit Care. 2024 Nov 25;28:387. doi: 10.1186/s13054-024-05159-9 (PMC11587605; doi:10.1186/s13054-024-05159-9)
Supplement: Supplementary file 1 — Additional file 1. [file 13054_2024_5159_MOESM1_ESM.docx]

**ADDITIONAL MATERIALS**

**Additional material 1: PRISMA checklist.**

**Additional material 2. Electronic search strategies.**

**Additional material 3. Detailed characteristics of included studies.**

**Additional material 4. Risk of bias of each study.**

**Additional material 5. Forest plots (subgroup analysis: different drug classes or devices).**

**Additional material 6. Forest plots (sensitivity analysis according to risk of bias).**

**Additional material 7. Forest plots (additional sensitivity analysis).**

**Additional material 8. Forest plots (additional secondary outcomes).**

**Additional material 9. Post-hoc analysis based on MDR bacteria detection.**

**Additional material 10. Additional data (overall duration of antibiotic treatment).**

**Additional material 11. Funnel plots.**

#

# Additional material 1. PRISMA checklist.

| **Section and Topic** | **Item #** | **Checklist item** | **Location where item is reported** |
| --- | --- | --- | --- |
| **TITLE** | | |  |
| Title | 1 | Identify the report as a systematic review. | 1 |
| **ABSTRACT** | | |  |
| Abstract | 2 | See the PRISMA 2020 for Abstracts checklist. | 2-3 |
| **INTRODUCTION** | | |  |
| Rationale | 3 | Describe the rationale for the review in the context of existing knowledge. | 4 |
| Objectives | 4 | Provide an explicit statement of the objective(s) or question(s) the review addresses. | 5 |
| **METHODS** | | |  |
| Eligibility criteria | 5 | Specify the inclusion and exclusion criteria for the review and how studies were grouped for the syntheses. | 6 |
| Information sources | 6 | Specify all databases, registers, websites, organisations, reference lists and other sources searched or consulted to identify studies. Specify the date when each source was last searched or consulted. | 6 |
| Search strategy | 7 | Present the full search strategies for all databases, registers and websites, including any filters and limits used. | 6 + Figure 1, AM 1-2 |
| Selection process | 8 | Specify the methods used to decide whether a study met the inclusion criteria of the review, including how many reviewers screened each record and each report retrieved, whether they worked independently, and if applicable, details of automation tools used in the process. | Figure 1,  AM 1-2 |
| Data collection process | 9 | Specify the methods used to collect data from reports, including how many reviewers collected data from each report, whether they worked independently, any processes for obtaining or confirming data from study investigators, and if applicable, details of automation tools used in the process. | 6-8 |
| Data items | 10a | List and define all outcomes for which data were sought. Specify whether all results that were compatible with each outcome domain in each study were sought (e.g. for all measures, time points, analyses), and if not, the methods used to decide which results to collect. | 6-8 |
|  | 10b | List and define all other variables for which data were sought (e.g. participant and intervention characteristics, funding sources). Describe any assumptions made about any missing or unclear information. | 6-8 |
| Study risk of bias assessment | 11 | Specify the methods used to assess risk of bias in the included studies, including details of the tool(s) used, how many reviewers assessed each study and whether they worked independently, and if applicable, details of automation tools used in the process. | 7-8 |
| Effect measures | 12 | Specify for each outcome the effect measure(s) (e.g. risk ratio, mean difference) used in the synthesis or presentation of results. | 9 |
| Synthesis methods | 13a | Describe the processes used to decide which studies were eligible for each synthesis (e.g. tabulating the study intervention characteristics and comparing against the planned groups for each synthesis (item #5)). | 8-9 |
|  | 13b | Describe any methods required to prepare the data for presentation or synthesis, such as handling of missing summary statistics, or data conversions. | 8-9 |
|  | 13c | Describe any methods used to tabulate or visually display results of individual studies and syntheses. | 8-9 |
|  | 13d | Describe any methods used to synthesize results and provide a rationale for the choice(s). If meta-analysis was performed, describe the model(s), method(s) to identify the presence and extent of statistical heterogeneity, and software package(s) used. | 8-9 |
|  | 13e | Describe any methods used to explore possible causes of heterogeneity among study results (e.g. subgroup analysis, meta-regression). | 8-9 |
|  | 13f | Describe any sensitivity analyses conducted to assess robustness of the synthesized results. | 8-9 |
| Reporting bias assessment | 14 | Describe any methods used to assess risk of bias due to missing results in a synthesis (arising from reporting biases). | 7 |
| Certainty assessment | 15 | Describe any methods used to assess certainty (or confidence) in the body of evidence for an outcome. | 8 |
| **RESULTS** | | |  |
| Study selection | 16a | Describe the results of the search and selection process, from the number of records identified in the search to the number of studies included in the review, ideally using a flow diagram. | 10 |
|  | 16b | Cite studies that might appear to meet the inclusion criteria, but which were excluded, and explain why they were excluded. | 10 |
| Study characteristics | 17 | Cite each included study and present its characteristics. | AM 3 |
| Risk of bias in studies | 18 | Present assessments of risk of bias for each included study. | Figure 2, AM 4 |
| Results of individual studies | 19 | For all outcomes, present, for each study: (a) summary statistics for each group (where appropriate) and (b) an effect estimate and its precision (e.g. confidence/credible interval), ideally using structured tables or plots. | 10-11, Table 1,  AM 5 |
| Results of syntheses | 20a | For each synthesis, briefly summarise the characteristics and risk of bias among contributing studies. | Figures,  Table 1, AM 4 |
|  | 20b | Present results of all statistical syntheses conducted. If meta-analysis was done, present for each the summary estimate and its precision (e.g. confidence/credible interval) and measures of statistical heterogeneity. If comparing groups, describe the direction of the effect. | Figures,  Table 1, AM  4-12 |
|  | 20c | Present results of all investigations of possible causes of heterogeneity among study results. | Figures,  Table 1, AM  4-12 |
|  | 20d | Present results of all sensitivity analyses conducted to assess the robustness of the synthesized results. | Figures,  Table 1, AM  4-12 |
| Reporting biases | 21 | Present assessments of risk of bias due to missing results (arising from reporting biases) for each synthesis assessed. | Figures  Table 1, AM  4-12 |
| Certainty of evidence | 22 | Present assessments of certainty (or confidence) in the body of evidence for each outcome assessed. | Figures,  Table 1, AM  4-12 |
| **DISCUSSION** | | |  |
| Discussion | 23a | Provide a general interpretation of the results in the context of other evidence. | 12-14 |
|  | 23b | Discuss any limitations of the evidence included in the review. | 13-14 |
|  | 23c | Discuss any limitations of the review processes used. | 13-14 |
|  | 23d | Discuss implications of the results for practice, policy, and future research. | 14 |
| **OTHER INFORMATION** | | |  |
| Registration and protocol | 24a | Provide registration information for the review, including register name and registration number, or state that the review was not registered. | 1 |
|  | 24b | Indicate where the review protocol can be accessed, or state that a protocol was not prepared. | 1 |
|  | 24c | Describe and explain any amendments to information provided at registration or in the protocol. | NA |
| Support | 25 | Describe sources of financial or non-financial support for the review, and the role of the funders or sponsors in the review. | NA |
| Competing interests | 26 | Declare any competing interests of review authors. | 15 |
| Availability of data, code and other materials | 27 | Report which of the following are publicly available and where they can be found: template data collection forms; data extracted from included studies; data used for all analyses; analytic code; any other materials used in the review. | NA |

**Additional material 2. Electronic Search Strategies.**

|  | PubMed | Scopus | Embase |
| --- | --- | --- | --- |
| 1 | ("Pneumonia"[Mesh]  OR "Pneumonia, Ventilator-Associated"[Mesh] | TITLE-ABS("Pneumonia”  OR "Pneumonia, Ventilator-Associated” | (*Pneumonia/  OR *Pneumonia, Ventilator-Associated/ |
| 1 | OR “pneumonia”[Title/Abstract]  OR “Pneumonias”[Title/Abstract]  OR “Pneumonitis”[Title/Abstract]  OR “Pneumonitides”[Title/Abstract]  OR “Experimental Lung”[Title/Abstract]  OR “Experimental lung”[Title/Abstract]  OR “Inflammations”[Title/Abstract]  OR “Inflammation, Experimental Lung”[Title/Abstract]  OR “Lung Inflammation, Experimental”[Title/Abstract]  OR “Lung Inflammations, Experimental”[Title/Abstract]  OR “Pulmonary Inflammation”[Title/Abstract]  OR “Inflammation, Pulmonary”[Title/Abstract]  OR “Inflammations, Pulmonary”[Title/Abstract]  OR “Pulmonary Inflammations”[Title/Abstract]  OR “Lung Inflammation”[Title/Abstract]  OR “Inflammation, Lung”[Title/Abstract]  OR “Inflammations, Lung”[Title/Abstract]  OR “Lung Inflammations”[Title/Abstract] | OR “pneumonia”  OR “Pneumonias”  OR “Pneumonitis”  OR “Pneumonitides”  OR “Experimental Lung”  OR “Experimental lung”  OR “Inflammations”  OR “Inflammation, Experimental Lung”  OR “Lung Inflammation, Experimental”  OR “Lung Inflammations, Experimental”  OR “Pulmonary Inflammation”  OR “Inflammation, Pulmonary”  OR “Inflammations, Pulmonary”  OR “Pulmonary Inflammations”  OR “Lung Inflammation”  OR “Inflammation, Lung”  OR “Inflammations, Lung”  OR “Lung Inflammations” | OR “pneumonia”.ab,kw,ti,ad.  OR “Pneumonias”.ab,kw,ti,ad.  OR “Pneumonitis”.ab,kw,ti,ad.  OR “Pneumonitides”.ab,kw,ti,ad.  OR “Experimental Lung”.ab,kw,ti,ad.  OR “Experimental lung”.ab,kw,ti,ad.  OR “Inflammations”.ab,kw,ti,ad.  OR “Inflammation, Experimental Lung”.ab,kw,ti,ad.  OR “Lung Inflammation, Experimental”.ab,kw,ti,ad.  OR “Lung Inflammations, Experimental”.ab,kw,ti,ad.  OR “Pulmonary Inflammation”.ab,kw,ti,ad.  OR “Inflammation, Pulmonary”.ab,kw,ti,ad.  OR “Inflammations, Pulmonary”.ab,kw,ti,ad.  OR “Pulmonary Inflammations”.ab,kw,ti,ad.  OR “Lung Inflammation”.ab,kw,ti,ad.  OR “Inflammation, Lung”.ab,kw,ti,ad.  OR “Inflammations, Lung”.ab,kw,ti,ad.  OR “Lung Inflammations”.ab,kw,ti,ad. |
| 1 | OR “ventilator associated pneumonia”[Title/Abstract]  OR “Ventilator-Associated Pneumonia”[Title/Abstract]  OR “VAP”[Title/Abstract]  OR “hospital acquired pneumonia”[Title/Abstract]  OR “HAP”[Title/Abstract]  OR “nosocomial pneumonia”[Title/Abstract]  OR “ICU-acquired pneumonia”[Title/Abstract]  OR “infection-related ventilator-associated  complications”[Title/Abstract]  OR “IVAC”[Title/Abstract]  OR “ventilator-associated respiratory  infections”[Title/Abstract]  OR “VARI”[Title/Abstract] | OR “ventilator associated pneumonia”  OR “Ventilator-Associated Pneumonia”  OR “VAP”  OR “hospital acquired pneumonia”  OR “HAP”  OR “nosocomial pneumonia”  OR “ICU-acquired pneumonia”  OR “infection-related ventilator-associated  complications”  OR “IVAC”  OR “ventilator-associated respiratory  infections”  OR “VARI” | OR “ventilator associated pneumonia”.ab,kw,ti,ad.  OR “Ventilator-Associated Pneumonia”.ab,kw,ti,ad.  OR “VAP”.ab,kw,ti,ad.  OR “hospital acquired pneumonia”.ab,kw,ti,ad.  OR “HAP”.ab,kw,ti,ad.  OR “nosocomial pneumonia”.ab,kw,ti,ad.  OR “ICU-acquired pneumonia”.ab,kw,ti,ad.  OR “infection-related ventilator-associated  complications”.ab,kw,ti,ad.  OR “IVAC”.ab,kw,ti,ad.  OR “ventilator-associated respiratory  infections”.ab,kw,ti,ad.  OR “VARI”.ab,kw,ti,ad. |
| 1 | OR “Artificial Respiration”[Title/Abstract]  OR “Artificial Respirations”[Title/Abstract]  OR “Respirations, Artificial”[Title/Abstract]  OR “Ventilation, Mechanical”[Title/Abstract]  OR “Mechanical Ventilations”[Title/Abstract]  OR “Ventilations, Mechanical”[Title/Abstract]  OR “Mechanical Ventilation”[Title/Abstract]) | OR “Artificial Respiration”  OR “Artificial Respirations”  OR “Respirations, Artificial”  OR “Ventilation, Mechanical”  OR “Mechanical Ventilations”  OR “Ventilations, Mechanical”  OR “Mechanical Ventilation”) | OR “Artificial Respiration”.ab,kw,ti,ad.  OR “Artificial Respirations”.ab,kw,ti,ad.  OR “Respirations, Artificial”.ab,kw,ti,ad.  OR “Ventilation, Mechanical”.ab,kw,ti,ad.  OR “Mechanical Ventilations”.ab,kw,ti,ad.  OR “Ventilations, Mechanical”.ab,kw,ti,ad.  OR “Mechanical Ventilation”.ab,kw,ti,ad.) |
|  | AND | AND | AND |
| 2 | ("Inhalation"[Mesh] | TITLE-ABS("Inhalation” | (*Inhalation/ |
| 2 | OR “inhaled”[Title/Abstract]  OR “inhalation”[Title/Abstract]  OR “Inhaling”[Title/Abstract]  OR “Inspiration, Respiratory”[Title/Abstract]  OR “Respiratory Inspiration”[Title/Abstract]  OR “aerolized”[Title/Abstract]  OR “aerolised”[Title/Abstract]  OR “aerosolized”[Title/Abstract]  OR “aerosolised”[Title/Abstract]  OR “nebulised”[Title/Abstract]  OR “nebulized”[Title/Abstract]  OR “nebulisation”[Title/Abstract]  OR “nebulization”[Title/Abstract]  OR “endotracheal”[Title/Abstract]  OR “endotracheally”[Title/Abstract]  OR “micronebulization”[Title/Abstract]  OR “micronebulisation”[Title/Abstract]) | OR “inhaled”  OR “inhalation”  OR “Inhaling”  OR “Inspiration, Respiratory”  OR “Respiratory Inspiration”  OR “aerolized”  OR “aerolised”  OR “aerosolized”  OR “aerosolised”  OR “nebulised”  OR “nebulized”  OR “nebulisation”  OR “nebulization”  OR “endotracheal”  OR “endotracheally”  OR “micronebulization”  OR “micronebulisation”) | OR “inhaled”.ab,kw,ti,ad.  OR “inhalation”.ab,kw,ti,ad.  OR “Inhaling”.ab,kw,ti,ad.  OR “Inspiration, Respiratory”.ab,kw,ti,ad.  OR “Respiratory Inspiration”.ab,kw,ti,ad.  OR “aerolized”.ab,kw,ti,ad.  OR “aerolised”.ab,kw,ti,ad.  OR “aerosolized”.ab,kw,ti,ad.  OR “aerosolised”.ab,kw,ti,ad.  OR “nebulised”.ab,kw,ti,ad.  OR “nebulized”.ab,kw,ti,ad.  OR “nebulisation”.ab,kw,ti,ad.  OR “nebulization”.ab,kw,ti,ad.  OR “endotracheal”.ab,kw,ti,ad.  OR “endotracheally”.ab,kw,ti,ad.  OR “micronebulization”.ab,kw,ti,ad.  OR “micronebulisation”.ab,kw,ti,ad.) |
|  |  |  |  |
|  | AND | AND | AND |
| 3 | ("Critical Care"[Mesh] | TITLE-ABS("Critical Care” | (*Critical Care/ |
| 3 | OR “Care, Critical”[Title/Abstract]  OR “Intensive Care”[Title/Abstract]  OR “Care, Intensive”[Title/Abstract]  OR “Surgical Intensive Care”[Title/Abstract]  OR “Care, Surgical Intensive”[Title/Abstract]  OR “Intensive Care, Surgical”[Title/Abstract] | OR “Care, Critical”  OR “Intensive Care”  OR “Care, Intensive”  OR “Surgical Intensive Care”  OR “Care, Surgical Intensive”  OR “Intensive Care, Surgical” | OR “Care, Critical”.ab,kw,ti,ad.  OR “Intensive Care”.ab,kw,ti,ad.  OR “Care, Intensive”.ab,kw,ti,ad.  OR “Surgical Intensive Care”.ab,kw,ti,ad.  OR “Care, Surgical Intensive”.ab,kw,ti,ad.  OR “Intensive Care, Surgical”.ab,kw,ti,ad. |
| 3 | OR “ICU”[Title/Abstract] | OR “ICU” | OR “ICU”.ab,kw,ti,ad. |
|  | OR “Critical Illnesses”[Title/Abstract]  OR “Critical Illness”[Title/Abstract]  OR “Illness, Critical”[Title/Abstract]  OR “Illnesses, Critical”[Title/Abstract]  OR “Critically Ill”[Title/Abstract]) | OR “Critical Illnesses”  OR “Critical Illness”  OR “Illness, Critical”  OR “Illnesses, Critical”  OR “Critically Ill”) | OR “Critical Illnesses”.ab,kw,ti,ad.  OR “Critical Illness”.ab,kw,ti,ad.  OR “Illness, Critical”.ab,kw,ti,ad.  OR “Illnesses, Critical”.ab,kw,ti,ad.  OR “Critically Ill”.ab,kw,ti,ad.) |

**Additional material 3: Detailed characteristics of included studies.**

| **Author, year** | **Journal** | **Type of study** | **Indication** | **Inclusion criteria** | **Main**  **exclusion criteria** | **Treatments (N)** | **Main**  **outcomes** | **Overall population (N)** | **Age (mean)** | **M (%)** |
| --- | --- | --- | --- | --- | --- | --- | --- | --- | --- | --- |
| **Niederman et al. (2012)** | Intensive Care Med | multicenter, randomized, placebo-  controlled,  double-blind, parallel-  group, phase II study | Therapeutic,safety study | mechanically ventilated adult patients with clinical diagnosis of  Gram-negative hospital-  acquired pneumonia, VAP or HCAP, with a Clinical Pulmonary Infection Score ≥ 6 and with at least one of four risk factors  for Gram-negative multidrug-  resistant organisms | Patients whose subsequent tracheal aspirate culture results showed only Gram-positive organisms or amikacin-resistant Gram-negative organisms (MIC ≥512 μg/mL); compromised immune function or immunosuppression; severe hypoxemia; positive end-expiratory pressure >15 cm H_2_O; cystic fibrosis, lung cancer, lung resection, bronchial obstruction, active tuberculosis, burns >40% of total body surface area; participation in another investigational study within the previous 28 days; receipt of any amikacin within the previous 14 days. | Amikacin inhaled 400 mg every 12h + IV antibiotics (16); Amikacin inhaled 400 mg every 24h with aerosol placebo + IV antibiotics (16);  placebo every 12h + IV antibiotics (16) | tracheal aspirate amikacin maximum concentration ≥ 6,400 lg/mL (25x 256 lg/mL reference minimum inhibitory  concentration) and a ratio of area under the aspirate concentration–  time curve (0–24 h) to minimum inhibitory concentration ≥100 on day 1 | 48 | 60.5 | 74 |
| **Palmer et al. (2014)** | Am J Respir Crit Care Med | double–blind placebo-controlled study | Therapeutic | mechanically ventilated adult patients with signs of respiratory infection (purulent secretions and Clinical Pulmonary Infection Score (≥ 6) | pregnancy, use of immunosuppressive agents except steroids,  neutrophil count <1000 WBC/mL), history of allergy to study drugs, primary diagnosis of  community acquired pneumonia | Aerosolized antibiotics + IV antibiotics (24);  saline placebo inhaled solution + IV antibiotics (18); | To assess efficacy of aerosolized antibiotics in eradicate MDRO in intubated patients without promoting  new resistance | 42 | 58.9 | 64 |
| **Liu et al. (2017)** | Chin Med J | single‑center, double‑blind study | Therapeutic | mechanically ventilated >48 h adult patients, with confirmed MDR‑GN bacteria VAP | pregnancy, perinatal  period, feeding period; history of allergy or adverse effect to amikacin or aerosolized therapy; acute or chronic renal insufficiency; airway obstructive factors or limitation; immunosuppression;  and requirement of small tidal volume MV | Amikacin inhaled + IV antibiotics (27);  saline placebo + IV antibiotics (25); | To assess efficacy and safety of amikacin aerosolized as an adjunctive therapy for VAP caused by MDR‑GNB | 52 | 66.4 | 61.5 |
| **Kollef et al (2017)** | Chest | randomized double-blind, placebo-  controlled, parallel group | Therapeutic | mechanically ventilated adult patients with confirmed clinical and micro-  biological  diagnosis of pneumonia, presence, or high suspicion, of GM bacteria | hypersensitivity to drugs used; IV antibiotics 72 h before randomization; PaO2/FiO2 ≤100 mmHg+diffuse chest radiograph infiltrates; refractory septic shock; flail chest, large pleural  effusions, lung cancer, lung abscess, bronchial obstruction, atypical/chemicalpneumonia,, cystic fibrosis, immunocompromised; creatinine >4 mg/dL; history of ototoxicity; hepatotoxicity; mechanical ventilation for >28 days; GCS 3; participation in other drug or device trial. | inhaled amikacin fosfomycin + IV meropenem or imipenem (71);  IV meropenem or imipenem (71); | To assess the change from  baseline in the Clinical Pulmonary Infection Score (CPIS) | 142 | 59.8 | 69.7 |
| **Ammar et al. (2018)** | Saudi J Anaesth | prospective, randomized, non-blinded,  controlled, single‑center trial | Therapeutic | mechanically ventilated >48h adult patients having VAP  with GN MDRO on sputum culture | pregnancy, immunosuppressive agents except for  steroids, neutrophil count ≤1000/mL,  allergy to the study drugs, primary  diagnosis of community‑acquired pneumonia,  renal impairment | IV amikacin + IV meropenem (30);  IV amikacin + IV meropenem + nebulized amikacin (30);  IV amikacin +  nebulized amikacin and meropenem (30); | To assess clinical outcome  of VAP | 90 | 55,3 | 75.5 |
| **Nassar et al. (2018)** | Egyptian Journal of Chest Diseases and Tuber-culosis | single-center, randomized, controlled study | Therapeutic | mechanically ventilated adult patients with confirmed clinical and microbiological  diagnoses of gram-negative VAP  . | creatinine clearance <30 ml/min, severe asthma, irreversible states of shock | inhaled colistin + IV antibiotics (52);  IV antibiotics (50); | To evaluate targeted microbiological and  clinical objective outcomes after a 5-day treatment  period | 102 | 55,5 | 75 |
| **Niederman et al. (2020)** | Lancet Infect Dis | prospective  double-  blind, randomized, placebo-controlled | Therapeutic | mechanically ventilated (>48h) adult patients with pneumonia diagnosed by chest radiography, and Clinical Pulmonary Infection Score (CPIS≥ 6). | systemic antibiotic treatment for  Gram-negative pneumonia for more than 48 h before the  study drug administration | inhaled amikacin  + IV antibiotics (262);  aerosolized saline placebo + IV antibiotics (263); | To assess the reducing mortality rate | 725 | 64 | 70.8 |
| **Hasan et al. (2021)** | J Crit Care Med | single-center, randomized study | Therapeutic | adult patients with MDR-Klebsiella pneumoniae VAP and less than two weeks of hospitalization before ICU  admission | any other bacterial growth in  endotracheal aspirates or in BAL fluid; surgery in the previous 3 months; chronic or end-stage renal diseases or  serum creatinine levels >2.5 mg/dL; 40 kg< body weight> 80 kg; APACHE II score >30; mild-to-severe hepatic impairment; hematological disorders; coinfection with other GN bacteria | IV Polymyxin B + nebulised polymyxin B (64);  IV Polymyxin  B alone (57); | To compare the use of  IV Polymyxin B with its nebulized form with IV polymyxin B alone | 121 | 64 | 57.8 |
| **Angermair et al. (2023)** | Anaesth Crit Care Pain Med | prospective, multicenter, double-blinded, randomized, placebo-controlled clinical trial | Therapeutic | mechanically ventilated adult patients with clinical and microbiological  diagnosis of gram-negative VAP | cystic fibrosis, colonization by non-fermenting organisms before hospitalization, having received antibiotics for more than 48 h before study drug  administration | Tobramicina inhaled + IV antibiotics (14); aerosolized saline placebo + IV antibiotics (12); | To evaluate eradication of the endo-bronchial  GN bacteria in the pulmonary system at visit 6 of the  treatment | 26 | 64.4 | 61.5 |
| **Maan et al. (2023)** | Cureus | double-blinded prospective  randomised control trial | Therapeutic | mechanically ventilated (>48h) adult patients with clinical and microbiological  diagnosis of gram-negative VAP | chest injuries, morbid obesity, uncontrolled comorbidities, hemodynamic instability, spine injuries, fungi,  GP bacteria in the endotracheal aspirate | IV + aerosolized  colistin (50);  aerosolized  colistin (50); | To compare the efficacy and safety of IV + aerosolized  colistin versus IV colistin alone in critically ill trauma patients | 100 | 55.6 | 71 |
| **Ghosh et al. (2024)** | Indian Journal of Critic al Care Medicine | randomized study | Therapeutic | mechanically ventilated adult patients with clinical and microbiological  diagnosis of gram-negative VAP or HAP | Patients requiring intravenous polypeptide antibiotics for other indication or those with known hypersensitivity to colistin of lack of clinical equipoise amongst treating clinicians or pregnant ladies or refusal of consent | aerosolized colistin 5MIU every 8h + IV polymyxin-B (30.000 UI/Kg of TBW) (11)); IV polymyxin-B alone (13) | Treatment success | 24 | 66 | 18 |

***Abbreviations:*** ICU, intensive care unit; MV, mechanical ventilation; VAP, ventilator-associated pneumonia; HAP, hospital acquired pneumonia; IV, intravenous; APACHE, acute physiologic assessment and chronic health evaluation; GN, gram-negative; MDRO, multi-drug resistant organisms; NA, no available; GCS, Glasgow Coma Scale; M, male.

**Additional material 4. Risk of bias of each study.
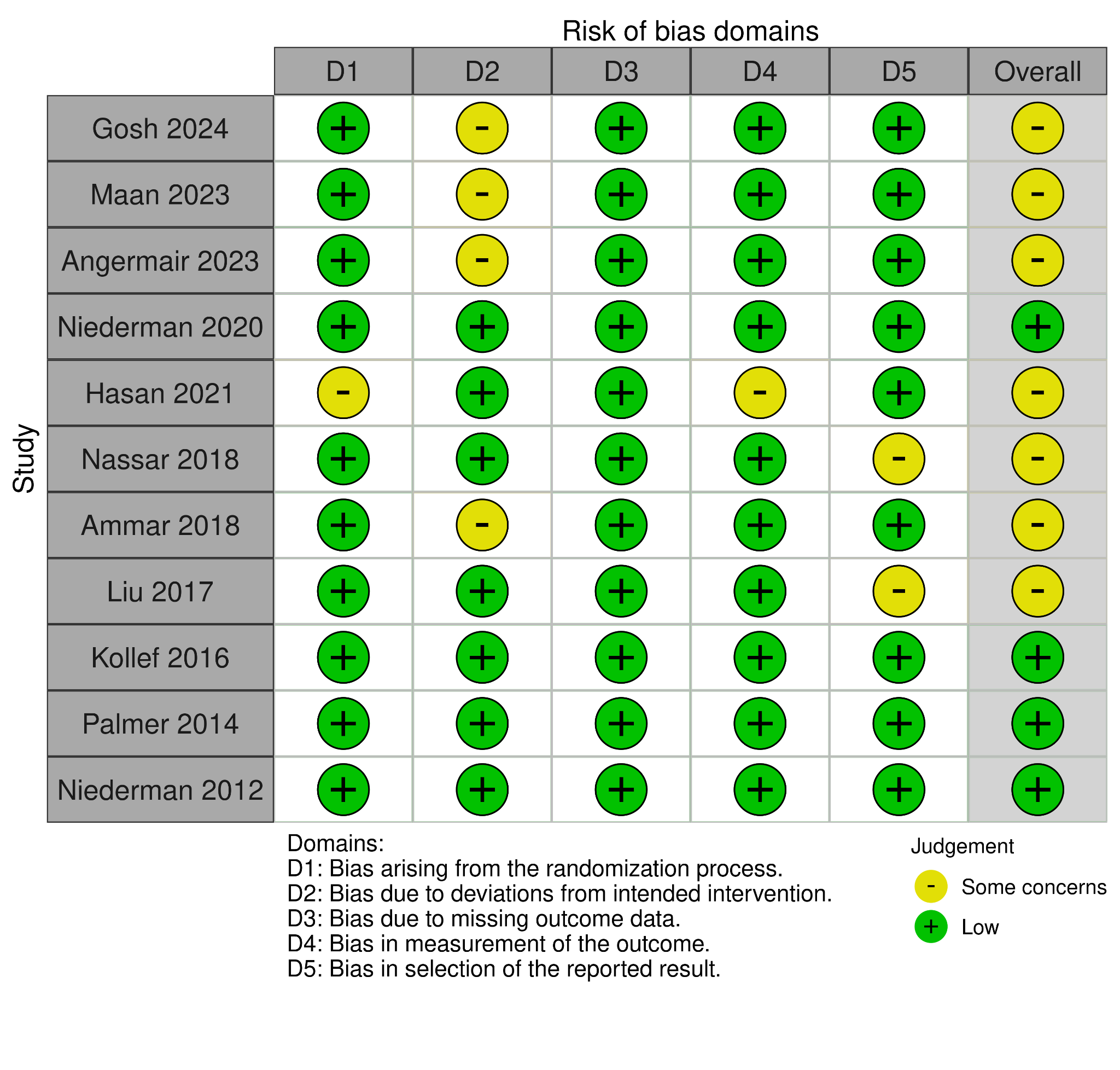
**

**Additional material 5. Forest plots (subgroup analysis).**

**A. According to different drug classes**

**
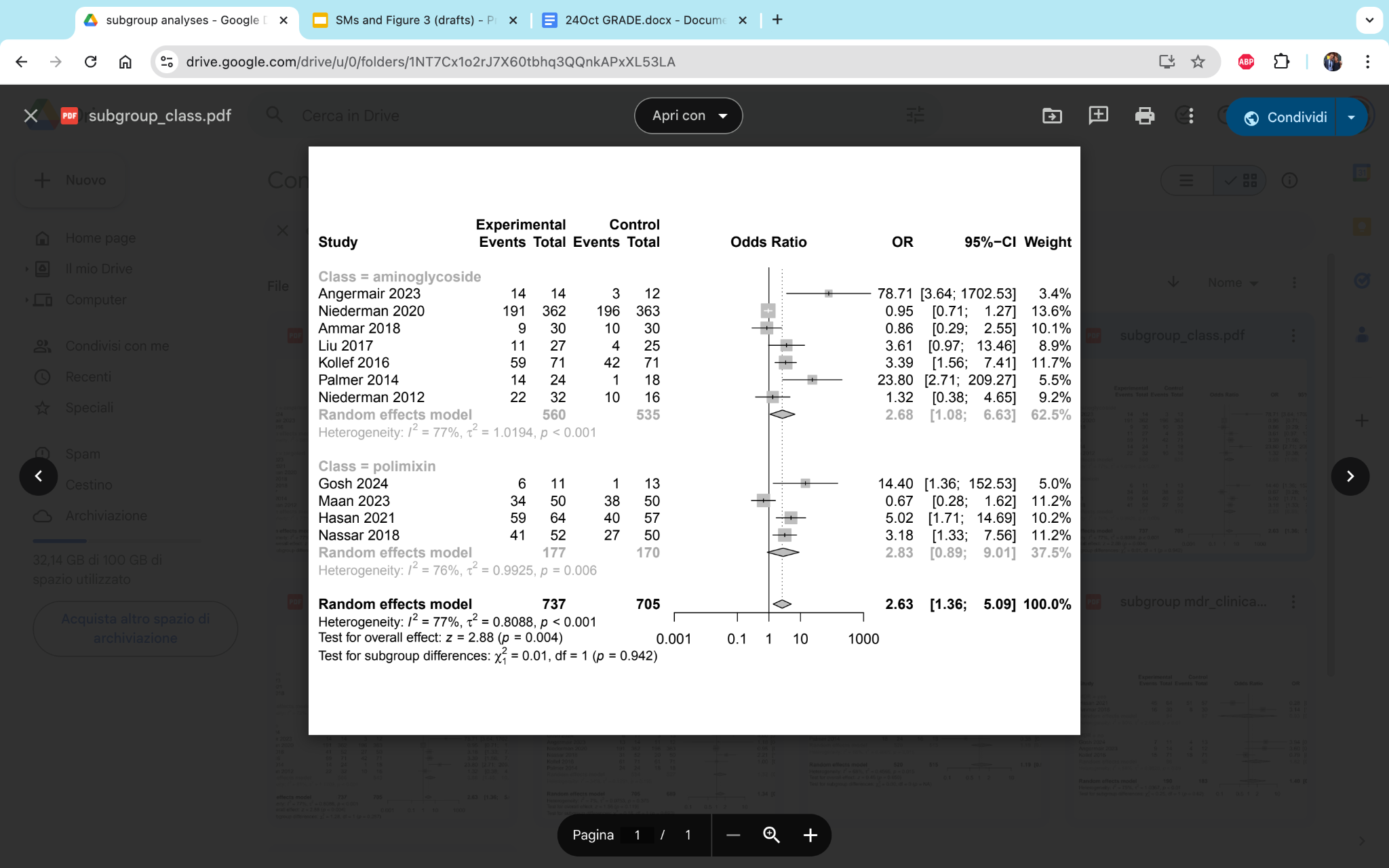
**

**B. According to different devices**

**
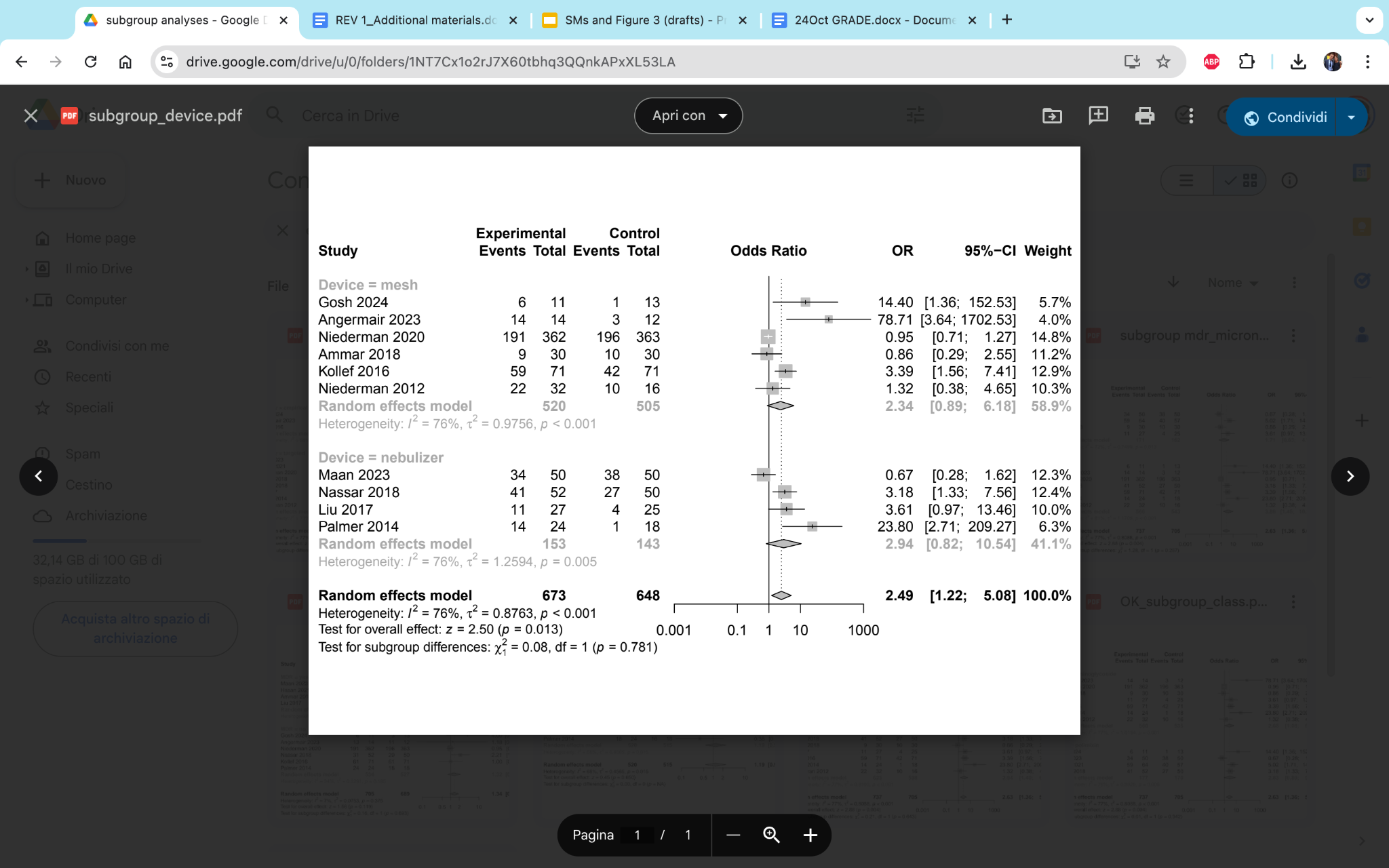
**

**Additional material 6. Forest plots (sensitivity analysis according to risk of bias).**

**
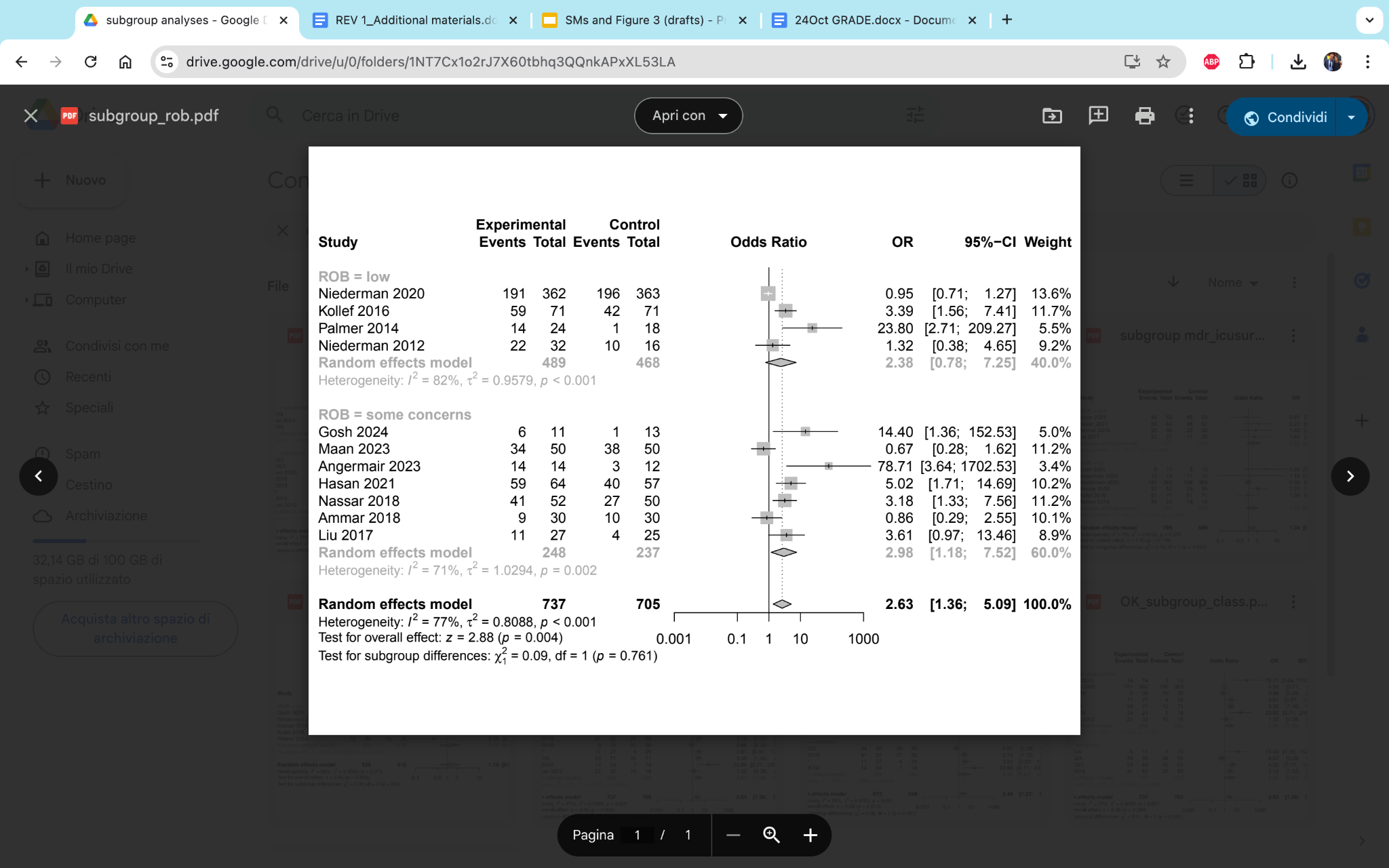
**

**Additional material 7. Forest plots (additional sensitivity analysis).**

1. **Removing one paper at a time.**

**
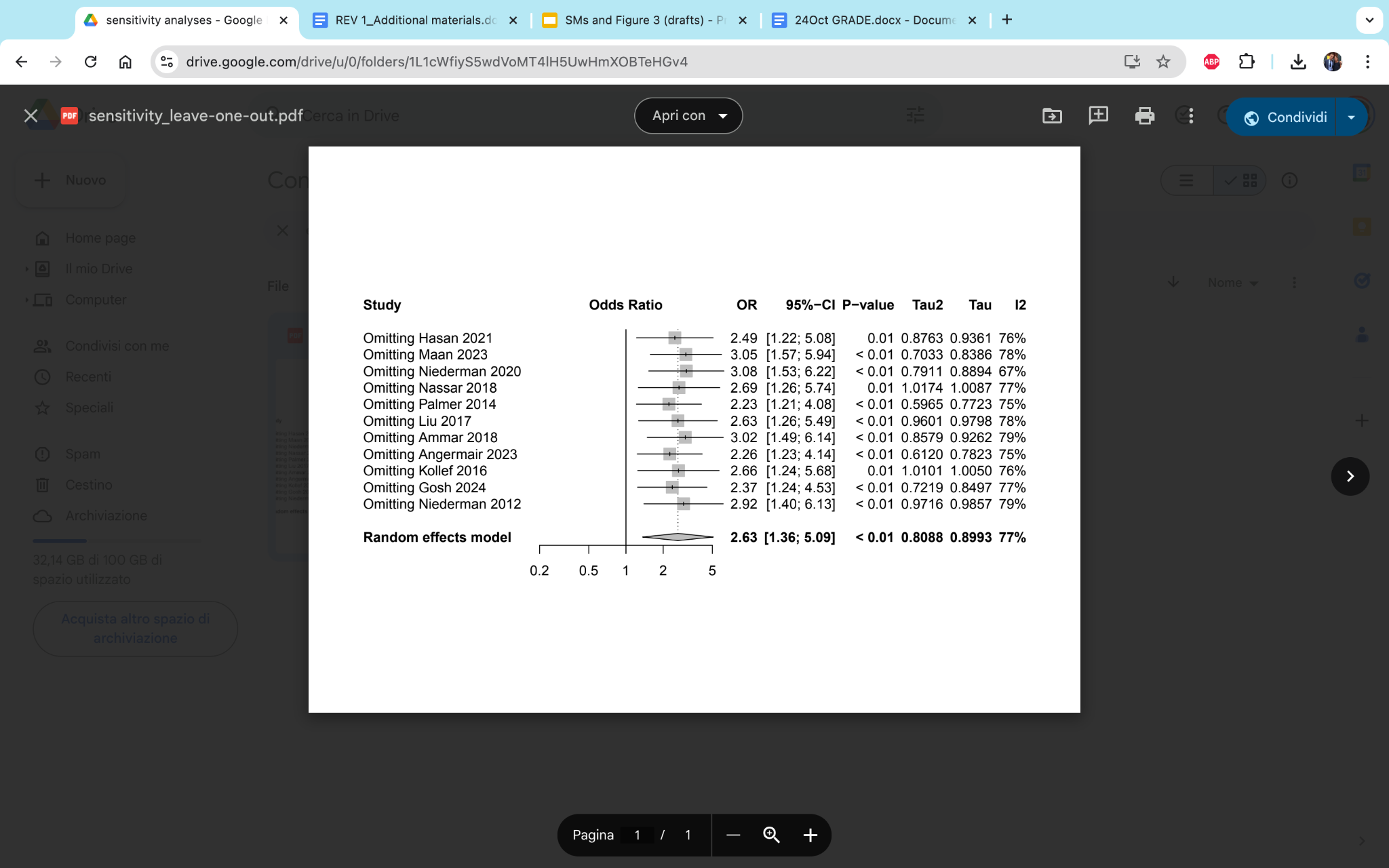
**

1. **Considering one of the two inhaled antibiotics groups at a time of the study by Ammar et al.**

**
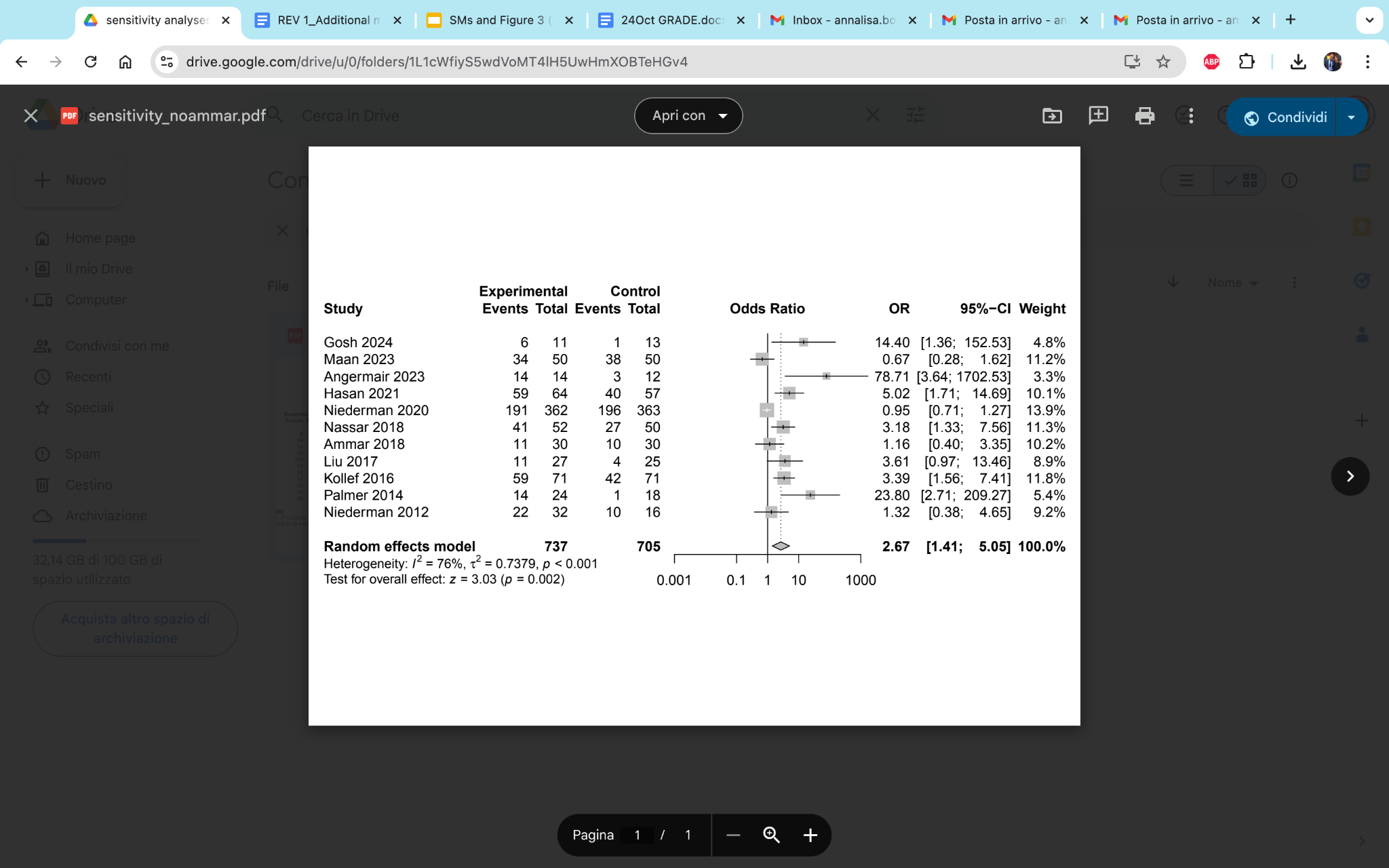
**

**Additional material 8. Forest plots (additional secondary outcomes).**

1. **Nephrotoxicity**

**
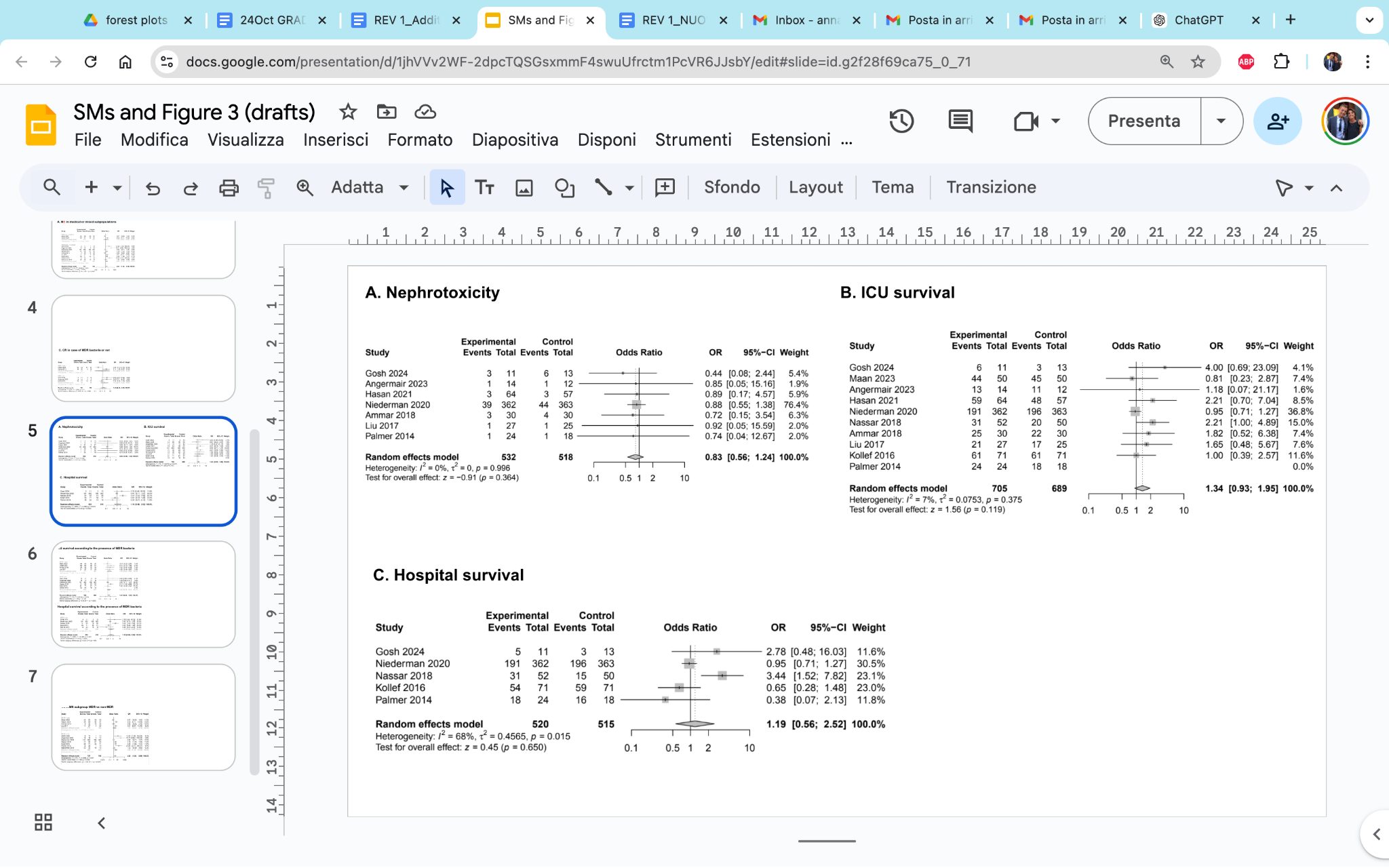
**

1. **ICU survival**

**
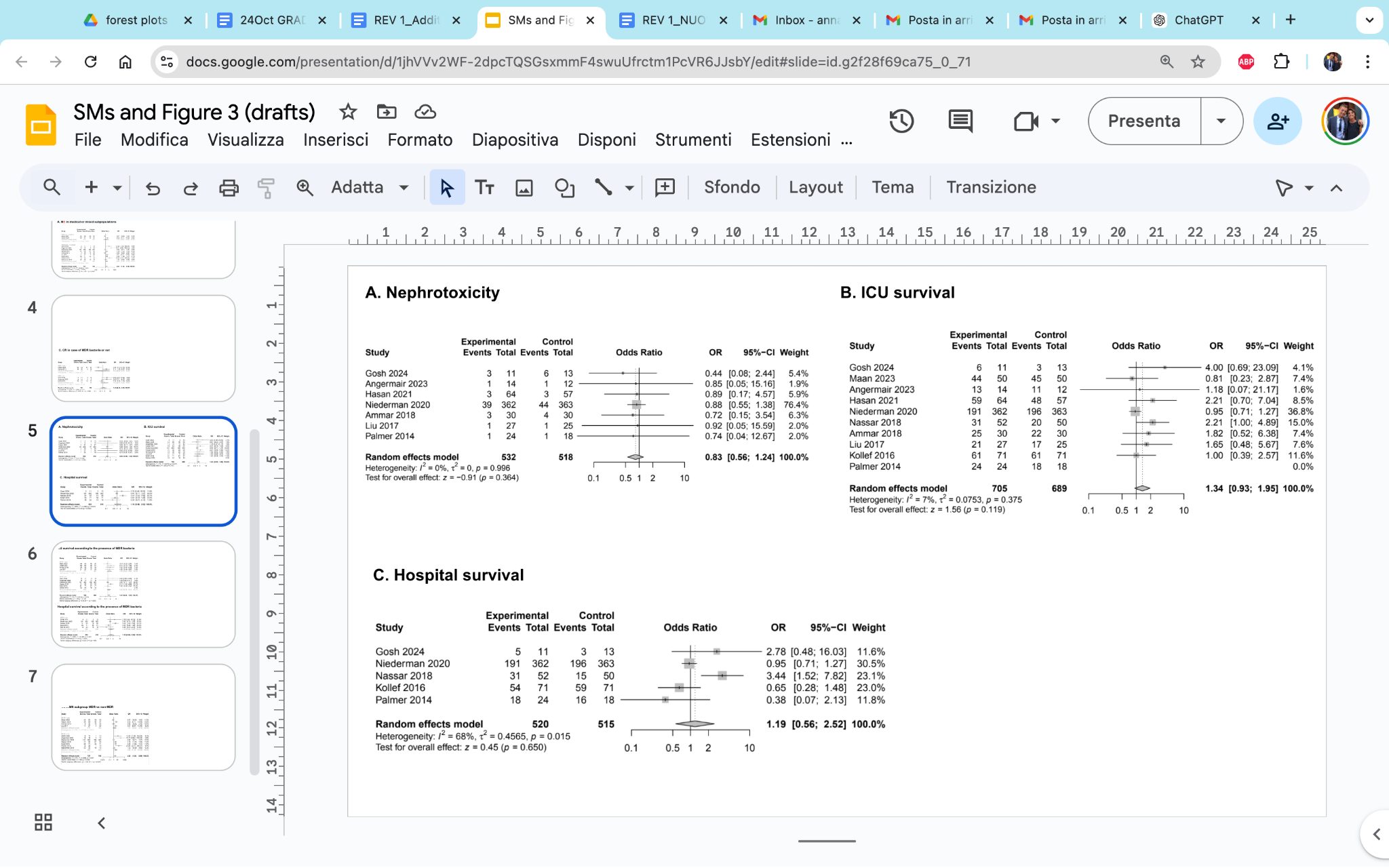
**

1. **Hospital survival**

**
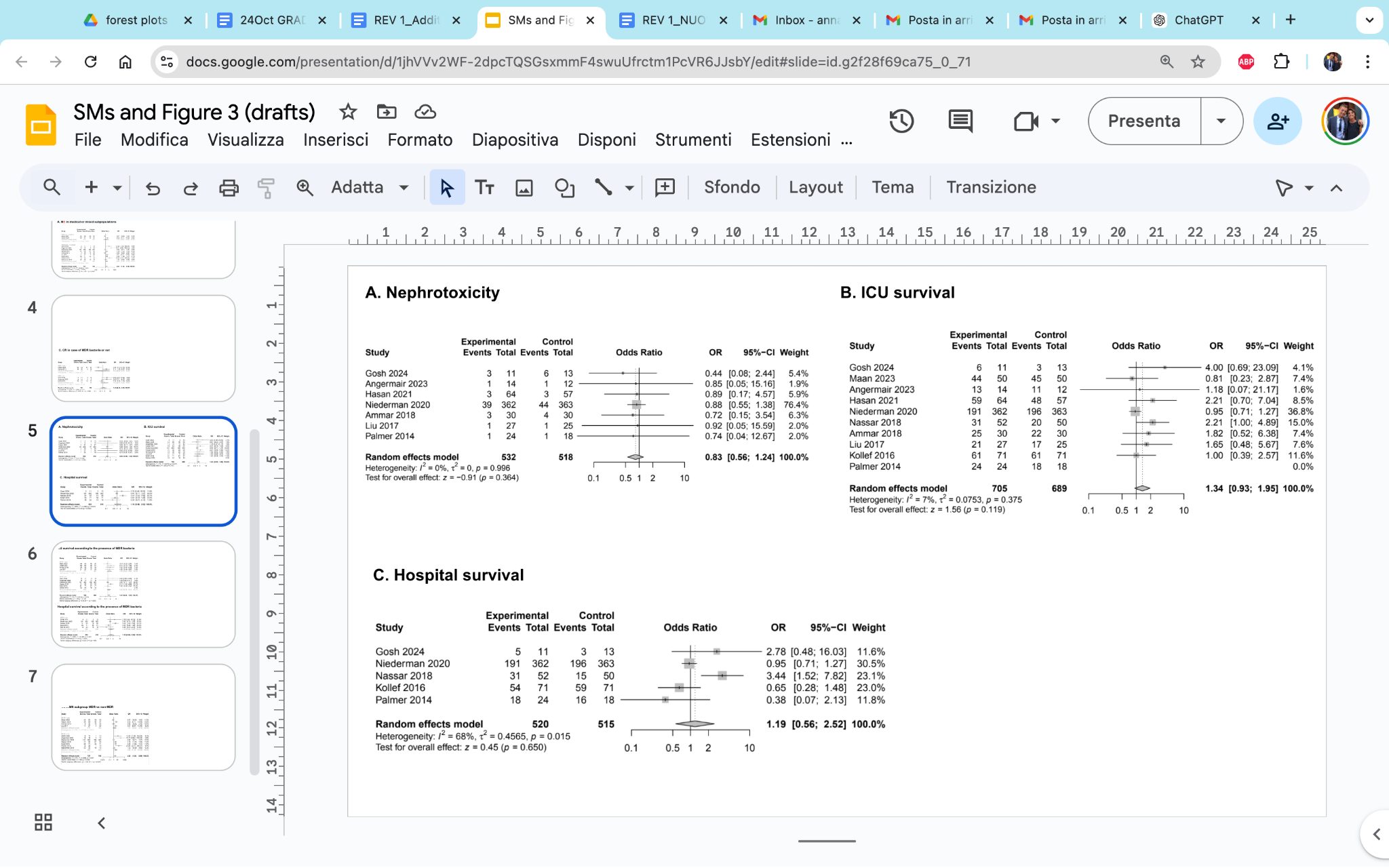
**

**Additional material 9. Post-hoc analysis based on MDR bacteria detection.**

*
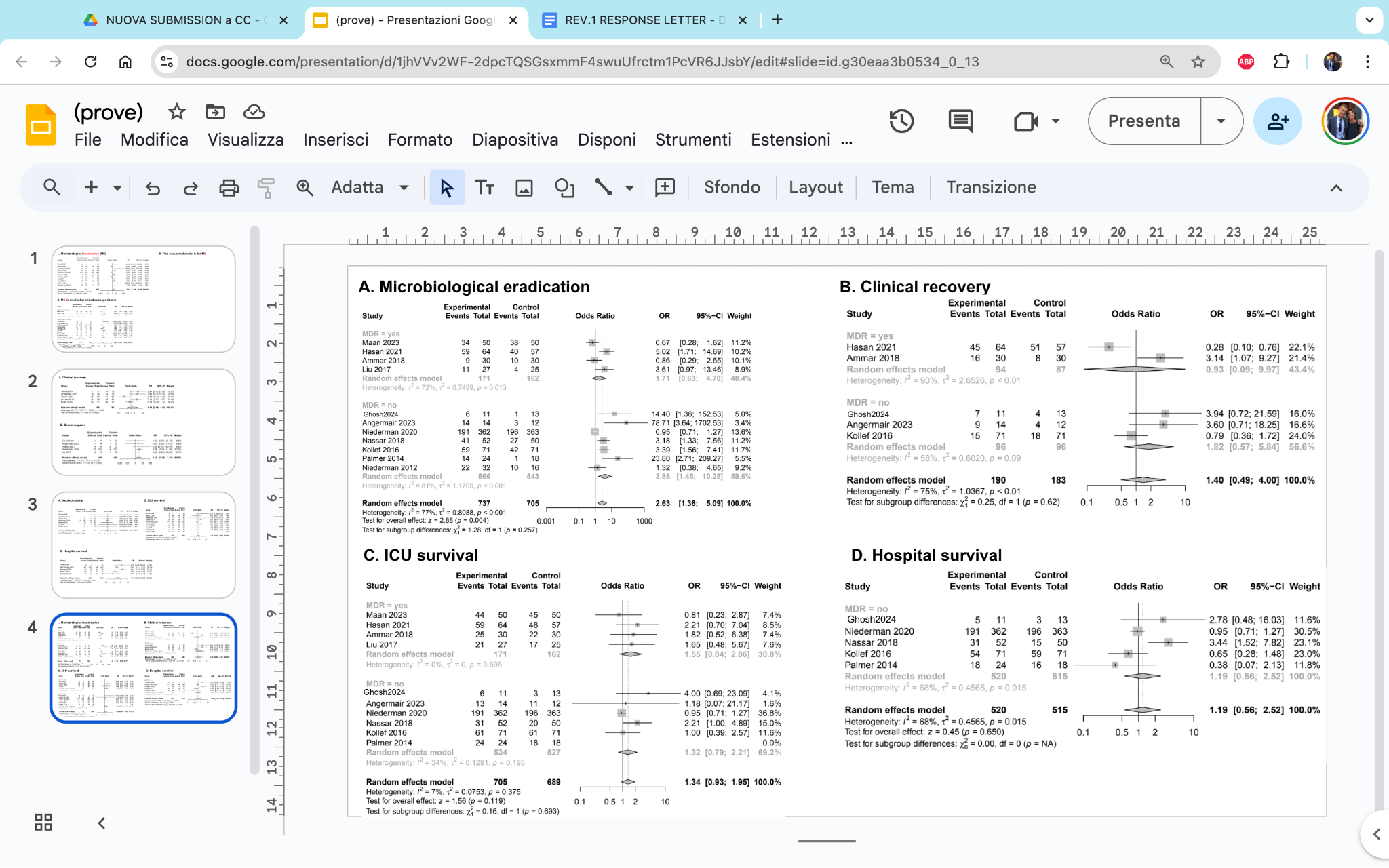
*

*
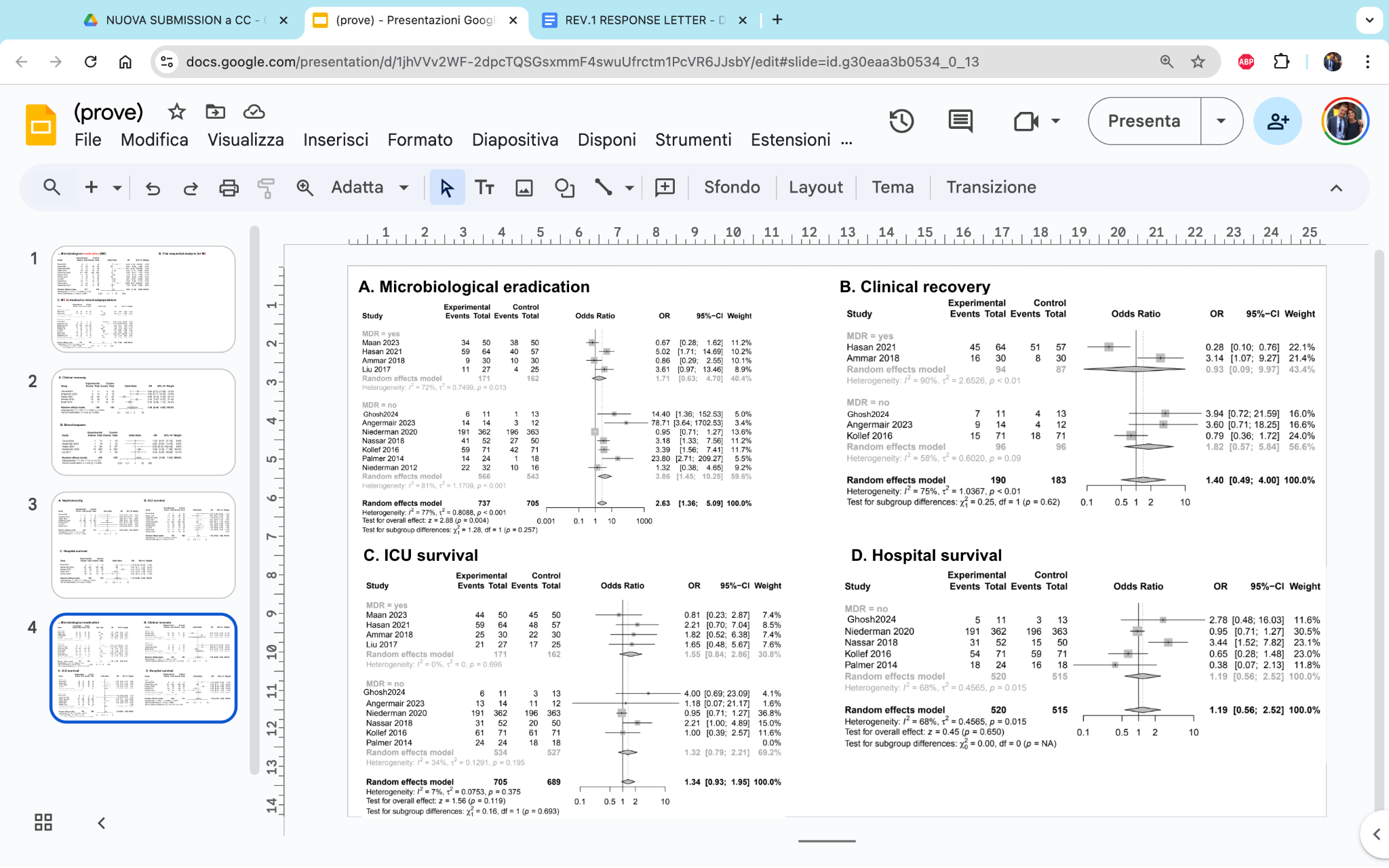
*

*
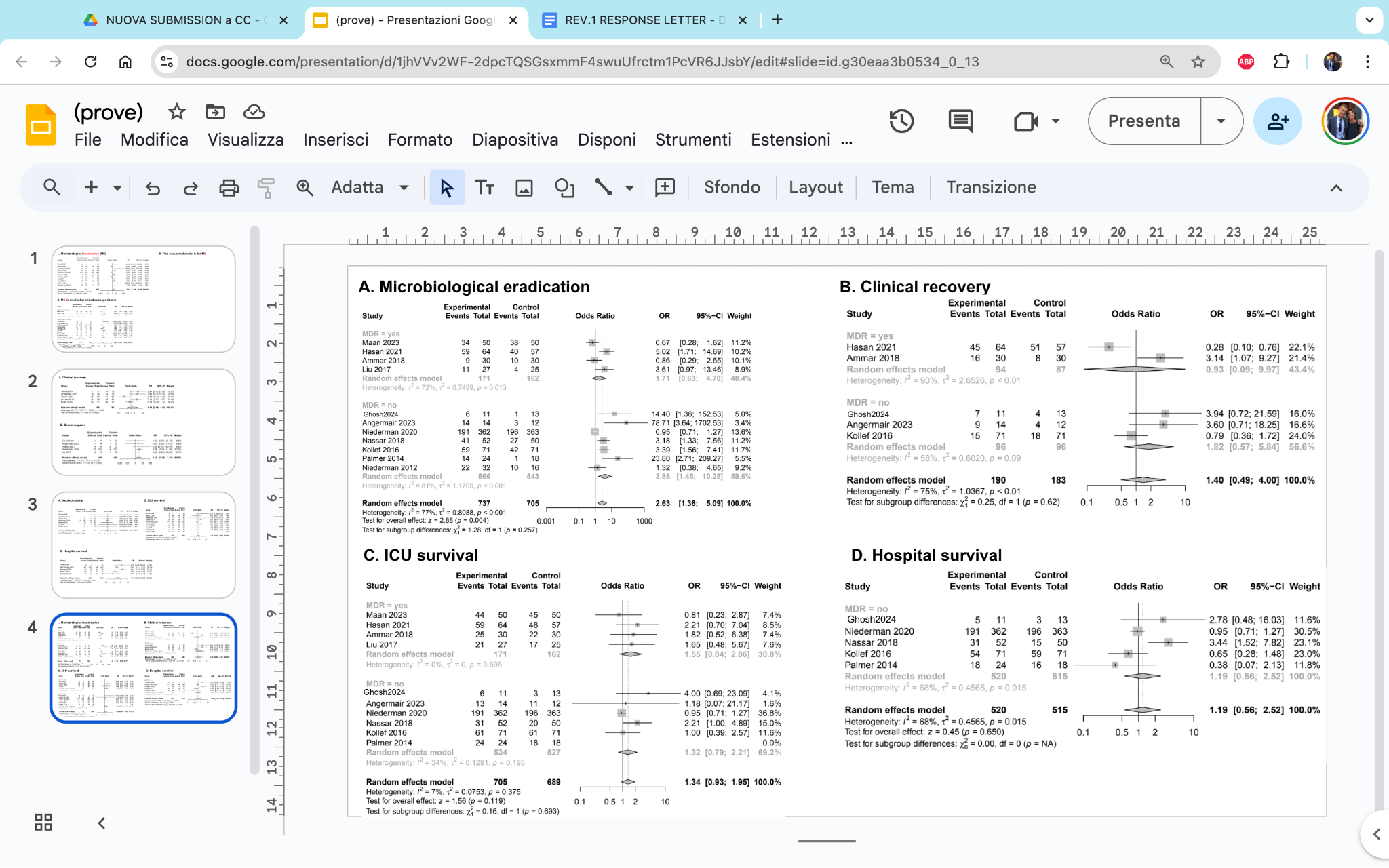
*

*
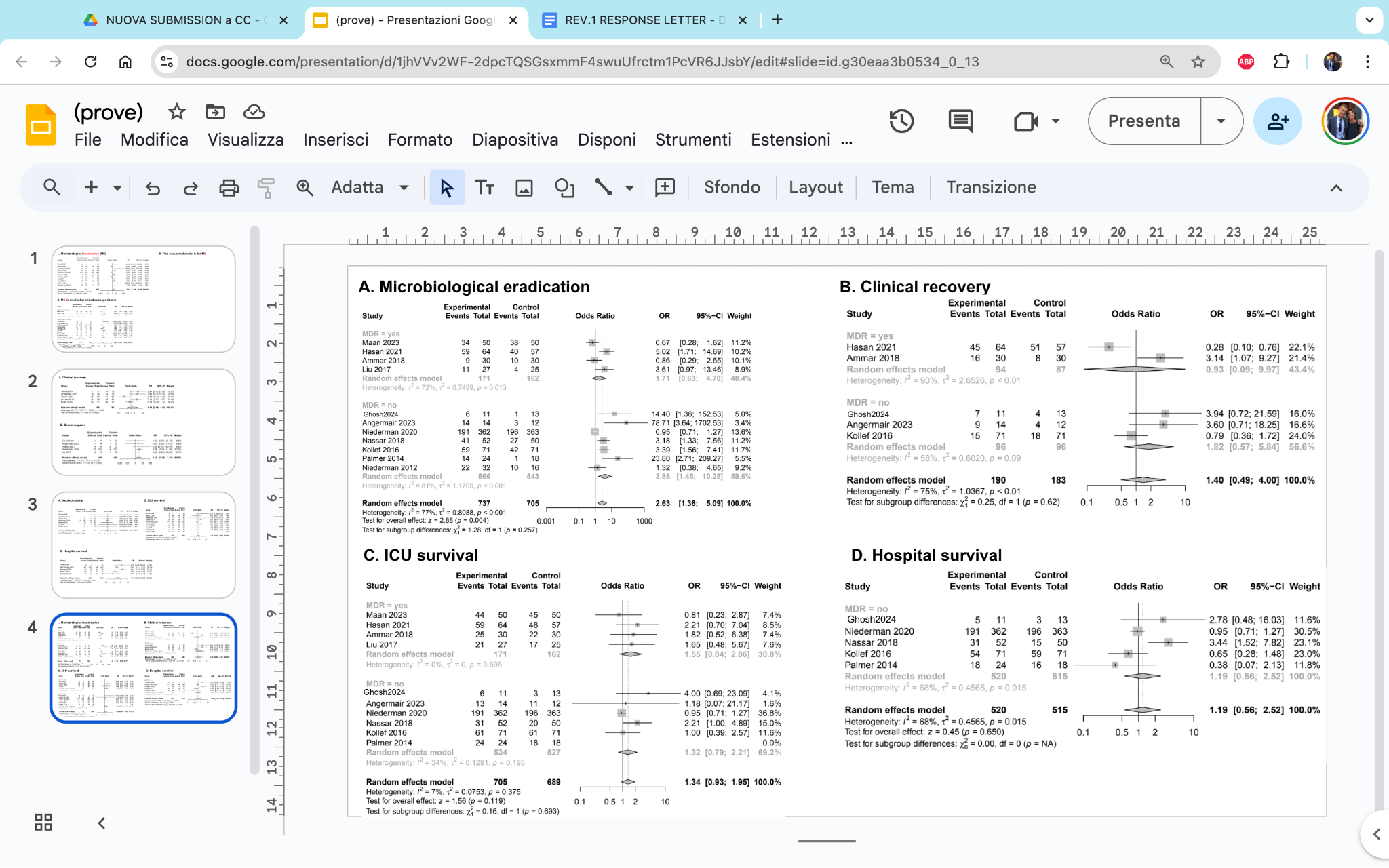
*

**Additional material 10. Additional data (overall duration of antibiotic treatment).**

*
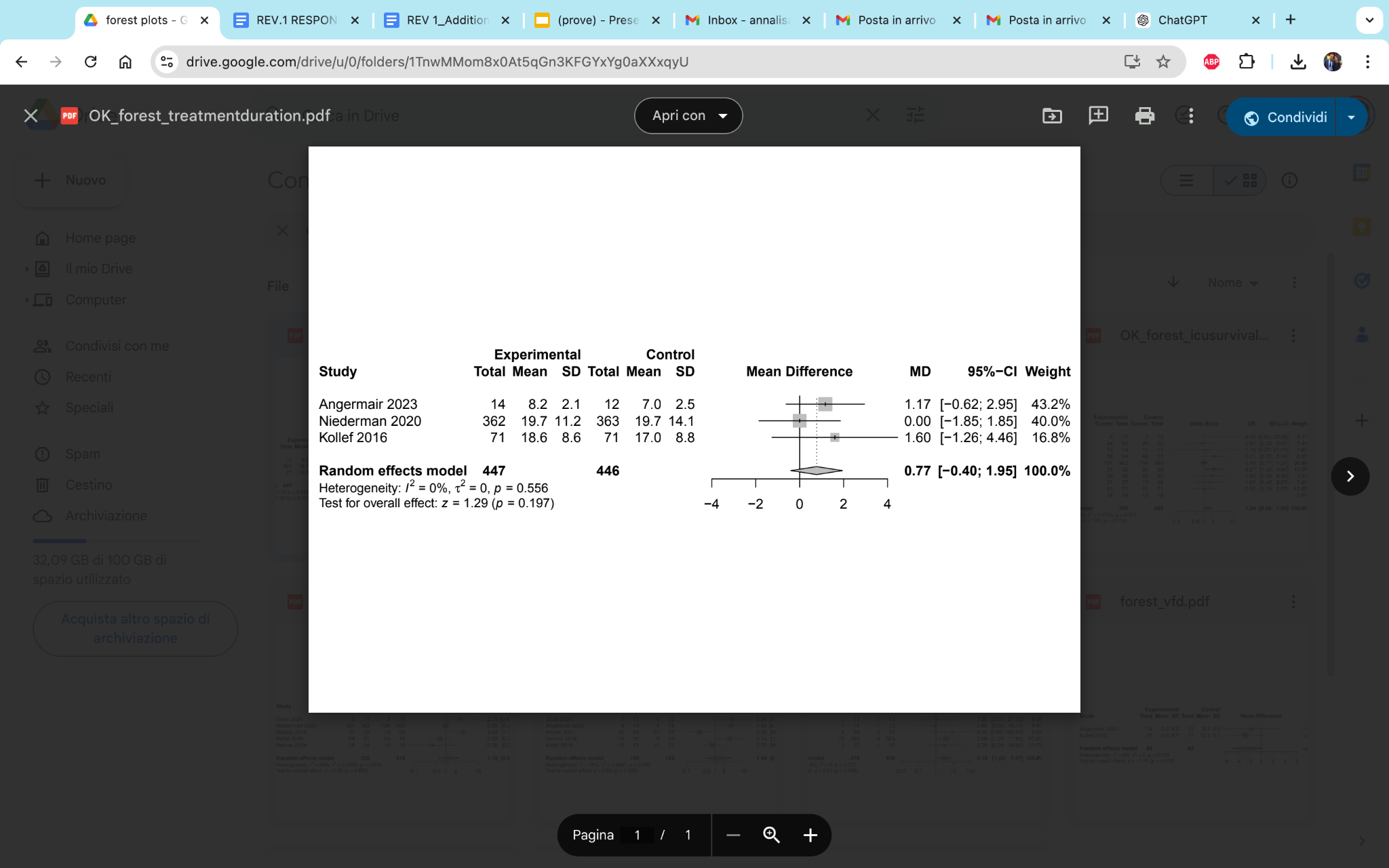
*

**Additional material 11. Funnel plots.**

| 1. **Microbiological eradication.** | 1. **Clinical recovery.** |
| --- | --- |
| **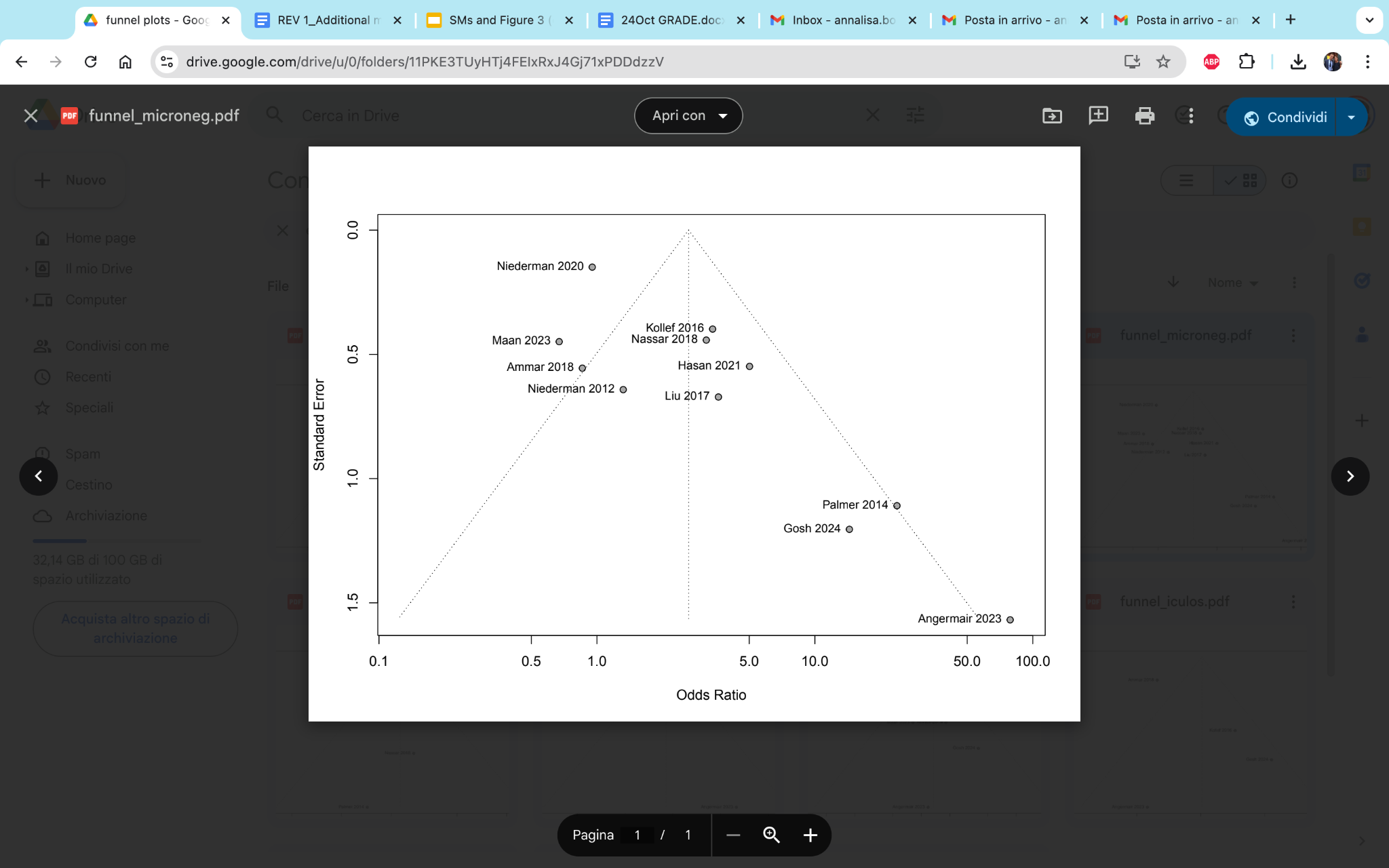** | **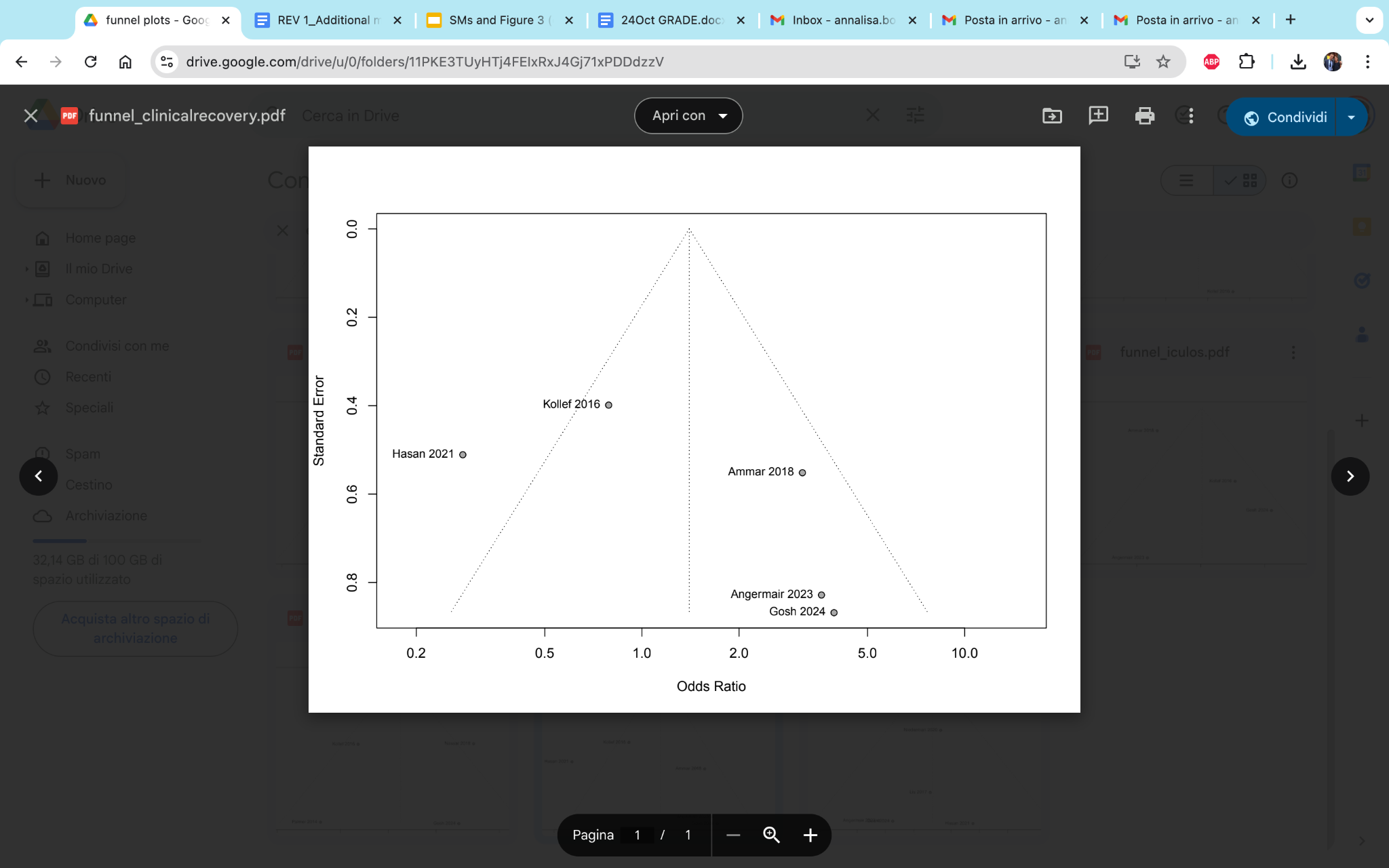** |
| 1. **Bronchospasm.** | 1. **Nephrotoxicity.** |
| **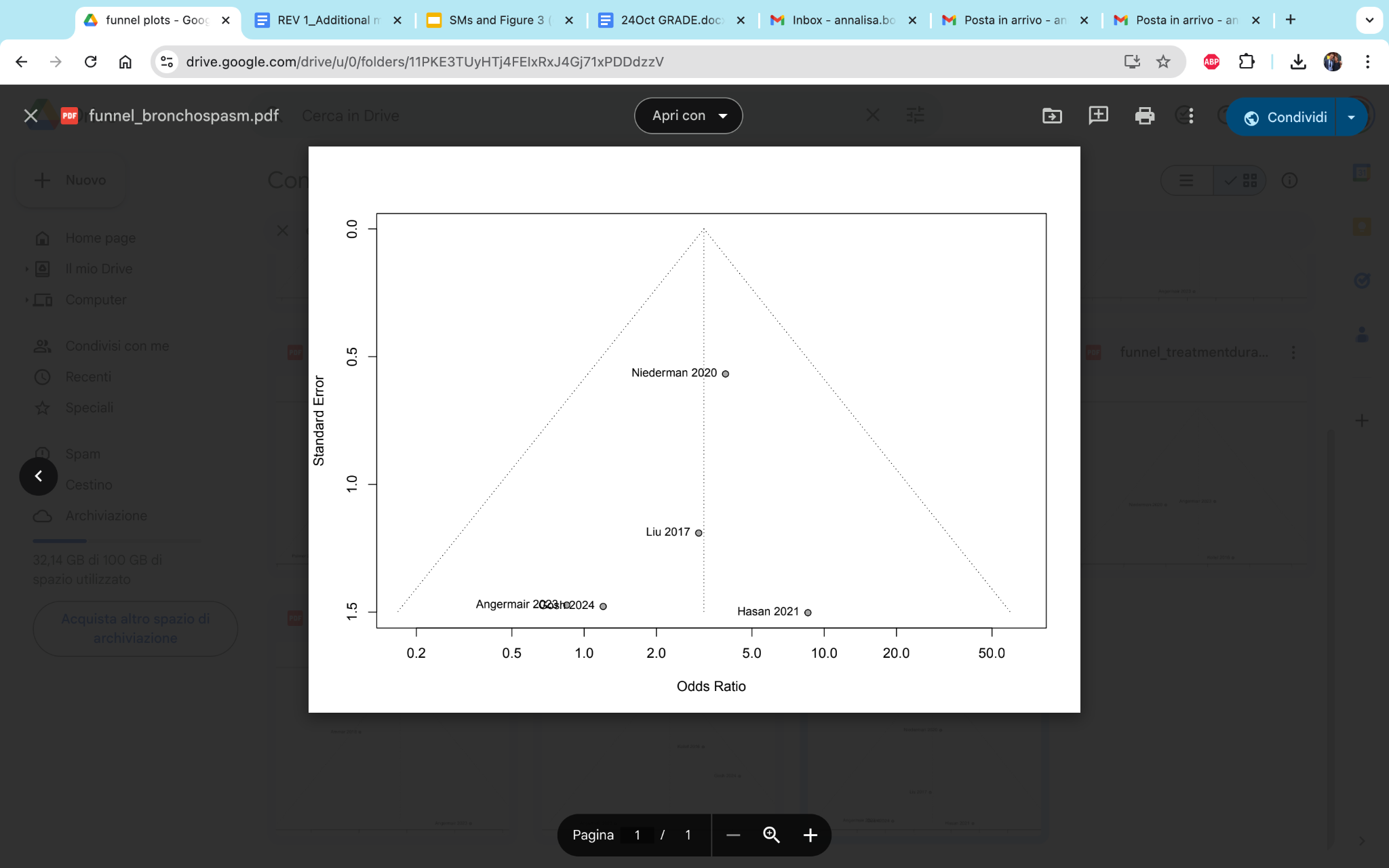** | **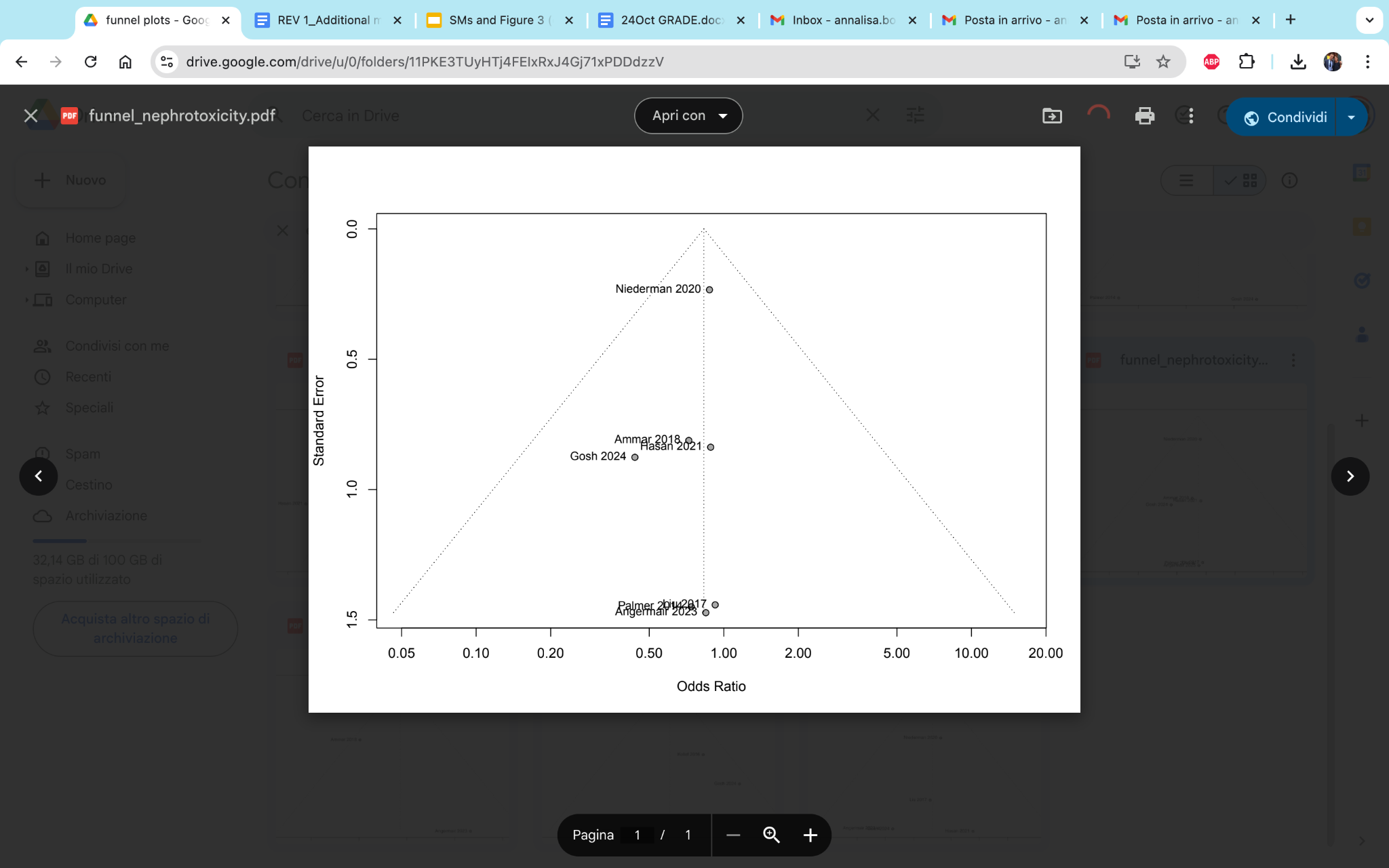** |
| 1. **ICU survival** | 1. **Hospital survival.** |
| **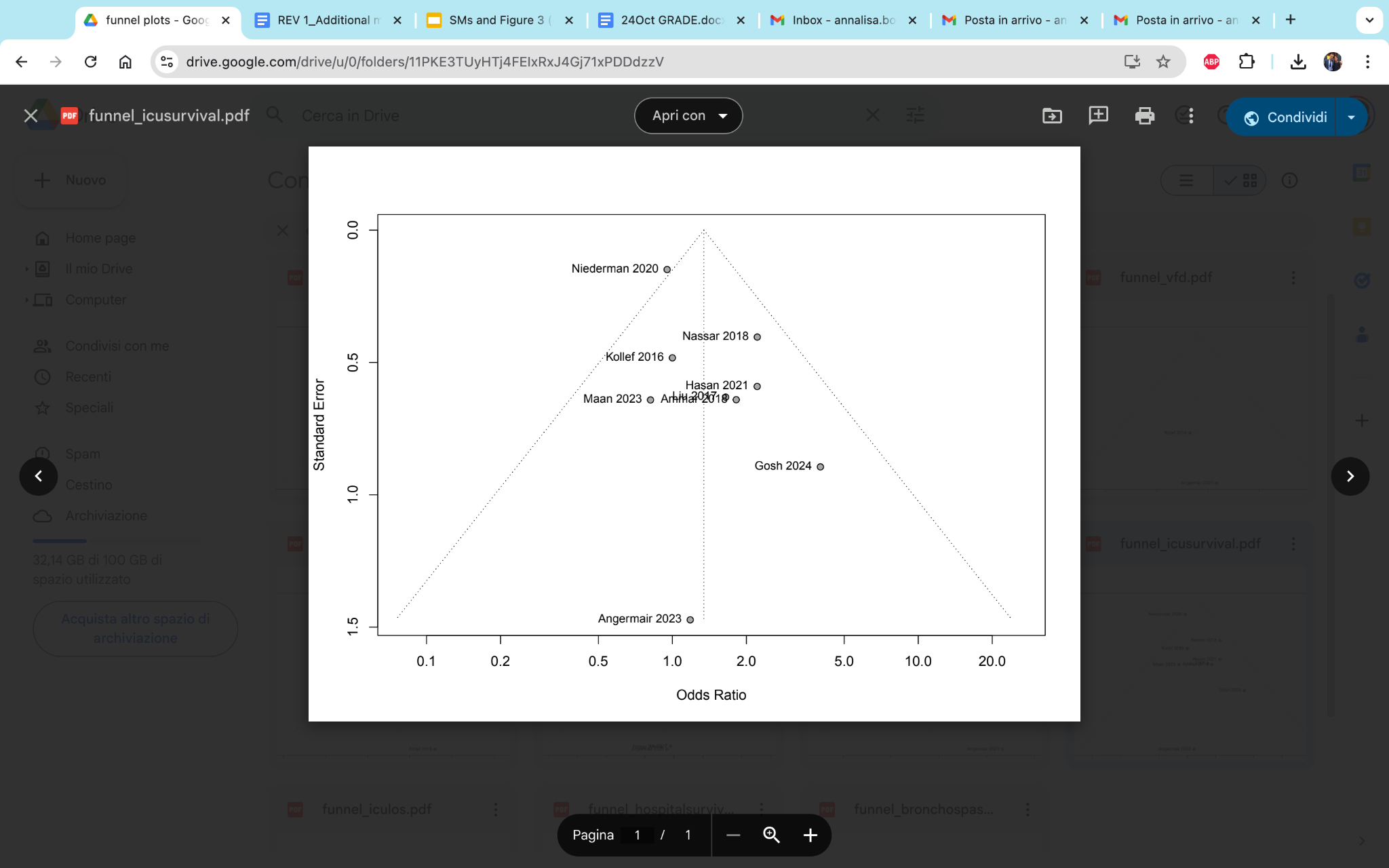** | **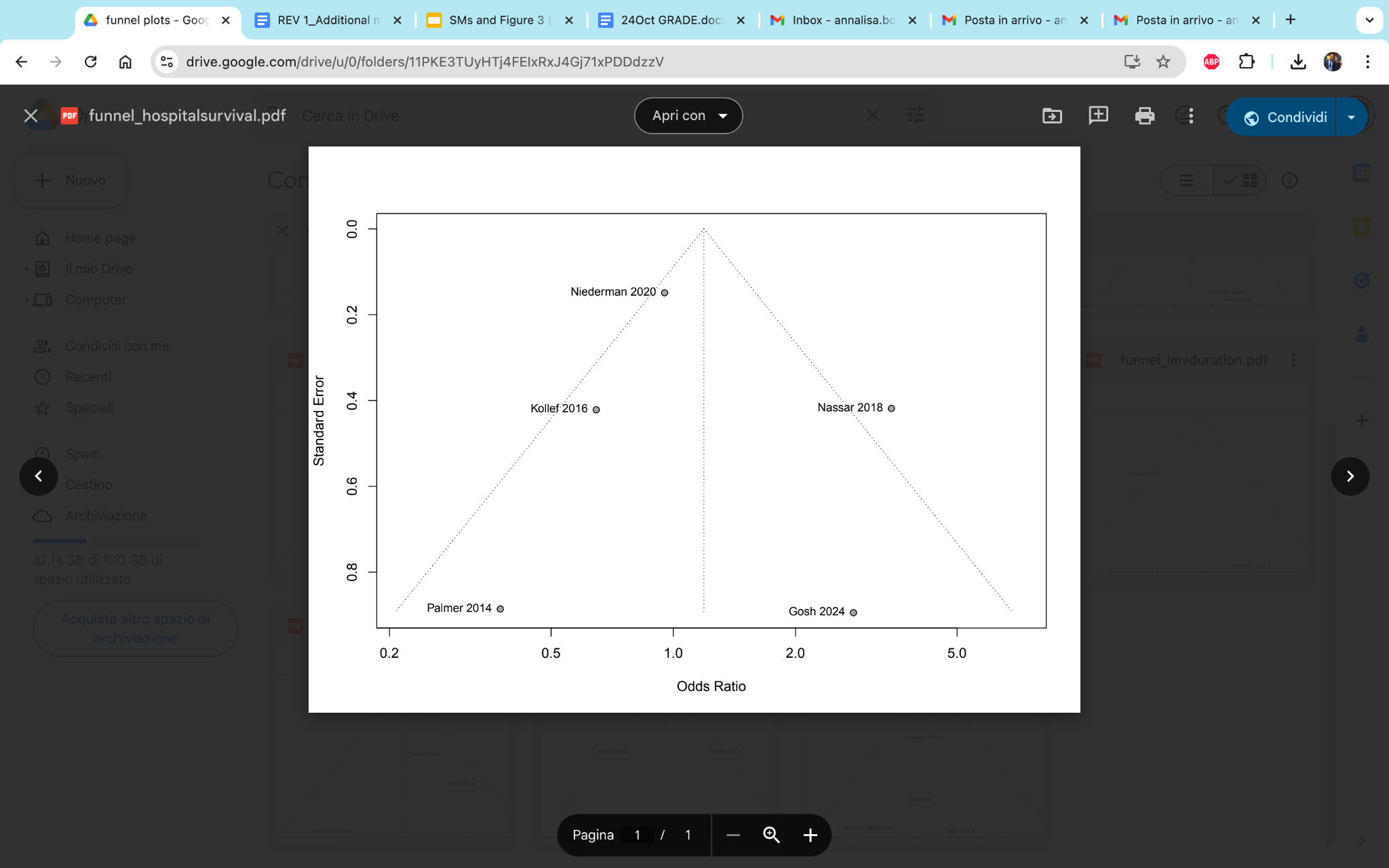** |
